# Niacinamide and its impact on stratum corneum hydration and structure

**DOI:** 10.1038/s41598-025-88899-0

**Published:** 2025-02-10

**Authors:** Thomas Sjöberg, Andebrhan Fsahaye, Emelie J. Nilsson, Silvia Letasiova, Itedale Namro, Lene Visdal-Johnsen, Nina Hrapovic, Sandra Smiljanic, Christina Österlund, Johan Engblom, Sebastian Björklund

**Affiliations:** 1 Department of Biomedical Sciences, Malmö University, Malmö, Sweden; 2 Biofilms Research Center for Biointerfaces, Malmö University, Malmö, Sweden; 3 MatTek In Vitro Life Science Laboratories, Bratislava, Slovakia; 4 CELLINK Bioprinting AB, Gothenburg, Sweden; 5 Global Research & Development, Oriflame Cosmetics AB, Stockholm, Sweden

**Keywords:** Skin barrier, Soft keratin, Niacinamide, Nicotinamide, Stratum corneum, X-ray diffraction, Water sorption isotherms, Biophysical chemistry, Biophysics

## Abstract

Niacinamide (NIA) is widely used in skincare for its favorable safety profile, anti-aging effects, and proven clinical efficacy in treating various skin conditions. However, its direct impact on the hydration and molecular organization of the stratum corneum (SC), the primary skin barrier, remains unclear. This study examines how NIA influences the SC’s lipid matrix organization, soft keratin structure, and water sorption behavior across varying relative humidity (RH) levels. Using small- and wide-angle X-ray diffraction and dynamic vapor sorption measurements, we compared NIA-treated SC samples to untreated controls under different RHs. The main findings show that while NIA is non-hygroscopic, it enhances water uptake of the SC at high humidity (95% RH). At low humidity (60% RH), NIA swells the keratin monomer spacing, although the SC water content remains low, suggesting a plasticizing effect that could increase SC flexibility in dry conditions. NIA also modifies the diffraction intensities from the lipid matrix differently at 60% and 95% RH, implying that it interacts with the SC lipid matrix and influences the water distribution within the SC lipid and protein domains. These effects appear independent of the investigated dose regime, indicating a specific concentration threshold. Overall, NIA shows distinct interaction with keratin, swelling the spacing between keratin monomers in dry conditions, without acting as a traditional keratolytic agent.

**Supplementary Information:**

The online version contains supplementary material available at 10.1038/s41598-025-88899-0.

## Introduction

The stratum corneum (SC) is the outermost layer of the skin and serves as the primary skin barrier, protecting against the entry of harmful substances and minimizing water loss^1^. Although very thin, measuring approximately 15 μm, the SC barrier properties are maintained because of the composite organization of non-viable cells, referred to as corneocytes, which are surrounded by a multilamellar lipid matrix^2^. The lipid matrix forms a continuous barrier element, ensuring minimal water diffusion from the inside and permeation of xenobiotics from the outside^3^. Its main lipid classes are long-chained and primarily saturated ceramides (CERs) and free fatty acids (FFAs), as well as cholesterol^4^. Due to the physicochemical properties of these lipids, the matrix is organized in a multilamellar phase with mainly crystalline hydrocarbon chains at ambient conditions^5^, representing a solid phase with very low diffusivity and solubility properties^3^.

Although the lipids are crucial for the SC barrier properties, they constitute merely about 5–15% of the total SC dry weight, while the corneocytes represent the main building block^6^. The corneocytes are packed with keratin fibrils, which are enclosed by the cornified cell envelope and an additional layer of covalently bound lipids^7–10^. The keratin fibrils of the SC are classified as soft keratin, characterized by a high abundance of disordered random coils, which contrasts the well-defined secondary structures of hard keratin found in nails and hair^11^. Keratin filaments play a crucial role in maintaining the structural integrity and overall elasticity of the SC. However, for keratin to become flexible, it needs to be hydrated. Therefore, to keep the skin soft and pliable, the SC tissue requires a continuous supply of water from the body, since environmental humidity cannot be controlled^12,13^. It has been suggested that 90–100% of the water taken up by the SC will preferentially partition inside the corneocytes, which implies that keratin is a major factor determining the SC hydration level and its capacity to hold water^14^. This is supported by electron microscopy studies showing that the thickness of corneocytes swell significantly upon hydration^2,15^. This suggests that the structural organization of the keratin filament network is crucial for the overall water sorption behavior of the SC. Therefore, investigations of how skin care ingredients affect the organization of keratin, in addition to their effects on lipid structure, are highly relevant.

To distinguish the effect of a specific skincare excipient from the overall effect of skin hydration, it is important to compare with untreated SC samples at the same relative humidity (RH)^16–18^. Also, given the complexity of skincare formulations that contain multiple ingredients, it is challenging to pinpoint the specific effects of individual substances. These issues are considered in the present study, where we use simple niacinamide (NIA) formulations to investigate how this popular skincare ingredient influences the SC molecular organization at different RHs. Specifically, we investigate how NIA affects the organization of the SC lipid matrix and the structural arrangement of soft keratin within corneocytes, as well as its impact on water sorption behavior under varying RH levels. To achieve this, we employ small- and wide-angle X-ray diffraction (SAXD and WAXD) to assess the structural organization of the SC, and dynamic vapor sorption (DVS) measurements to analyze its hydration process.

NIA, also known as nicotinamide, is a small polar molecule (MW 122 g/mol, logD − 0.4 at both pH 5.0 and 7.4), classified as a form of vitamin B3. It is widely used in topical skincare products due to its diverse benefits and safety profile^19^. It is established that NIA reduces transepidermal water loss and improves skin hydration and pliability^20,21^. These findings can be connected to both in vitro and in vivo studies, showing that NIA upregulates the biosynthesis of sphingolipids, including CERs and their precursors, as well as FFAs, and cholesterol^21^. NIA has also anti-inflammatory properties, benefiting conditions like acne, rosacea, and atopic dermatitis^22^. Furthermore, NIA exhibits antioxidant effects and can reduce DNA damage upon external insult^19,23–25^. It also inhibits the transfer of melanosomes to keratinocytes, thereby reducing hyperpigmentation and improving skin tone^26^. Clinical studies have demonstrated that NIA can reduce fine lines, wrinkles, and improve skin elasticity^19,20,22,27,28^. In summary, due to its beneficial properties, NIA is used for managing various skin disorders, including dermatitis, melasma, and actinic keratosis^20,28^. However, investigations of the effect of NIA on the molecular structure of the SC, as well as its effect on the water sorption behavior of the SC, are lacking. Therefore, the aim of this study is to examine how NIA influences the organization of the SC lipid matrix and soft keratin structures using SAXD and WAXD, as well as its effect on the SC hydration process via DVS measurements.

## Results

To investigate the effects of NIA on the hydration properties and molecular structure of the SC, we designed treatment protocols to evaluate the impact of pH and salinity variations. The physiological pH of the SC typically ranges from 4 to 6^29,30^. However, from a pharmaceutical/cosmetical perspective, the pH of skincare formulations can vary, influencing the local skin pH after topical application. To account for this, pretreatments were conducted using acidic (pH 5.0) and neutral (pH 7.4) buffers. Salinity is another critical factor influencing the SC properties, as prior studies have demonstrated that salt enhances its water sorption capacity^31,32^. However, little is known about how salinity affects the molecular organization of the SC. Importantly, both pH and salinity may influence the barrier properties of the SC, thereby affecting NIA penetration into the skin barrier and its subsequent effects on the investigated SC properties. Based on this rationale, our experimental design included pretreatment with citrate buffer at pH 5.0, with salt (CBS) and without additional salt (CB), and phosphate buffer at pH 7.4, with salt (PBS) and without additional salt (PB). The SC samples were incubated with 5 wt% NIA for 24 h, while untreated samples served as controls (i.e., treated in corresponding buffers without NIA). The selected NIA concentration reflects its typical concentration used in commercial formulations, although both lower and higher concentrations are also commonly employed^19,20^.

### Niacinamide is non-hygroscopic but increases the water uptake at high relative humidity

**Fig. 1:**
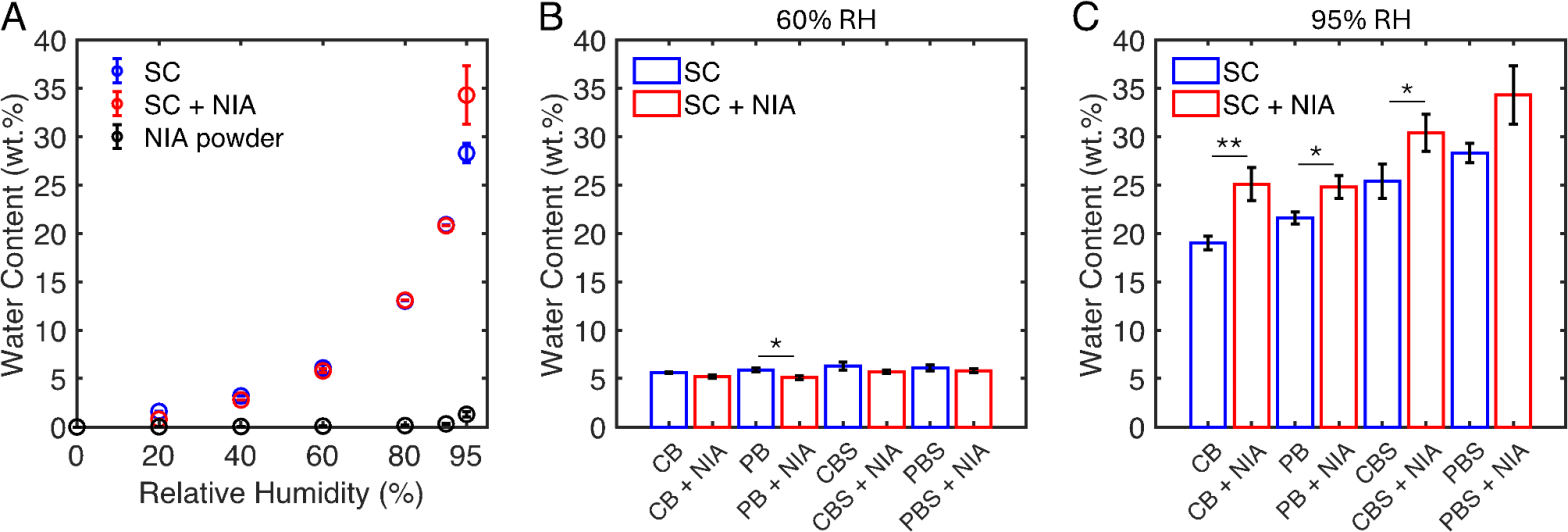
(**A**) Representative water sorption isotherms showing the water content as a function of relative humidity (RH) for untreated (SC) and NIA-treated samples (SC + NIA). These data correspond to pretreatment of SC in PBS (pH 7.4, with/without 5 wt% NIA), while data corresponding to all four buffers used as pretreatment media are compiled in Fig. S1. For comparison, the sorption isotherm of NIA in powder form is also included in (**A**). Panels (**B**) and (**C**) show the SC water content at 60% RH and 95% RH, respectively, for SC samples treated in acidic or neutral buffers, with or without added salt, and with or without 5 wt% NIA (*n =* 5). Abbreviations of pretreatment buffer media: CB – citrate buffer (pH 5.0), CBS – citrate buffer saline (pH 5.0), PB – phosphate buffer (pH 7.4), PBS – phosphate buffer saline (pH 7.4).

Representative results from the DVS experiments, which assesses the SC water content across varying RH levels, are presented in Fig. 1A. These data correspond to pretreatment of SC in PBS (pH 7.4), while the data corresponding to all four buffers used as pretreatment media are compiled in Fig. S1. In general, the SC water sorption isotherms exhibit a continuous water uptake across the entire RH range. Below 60% RH, the relationship between water uptake and RH is relatively linear, while the isotherms becomes steeper beyond this point, indicating higher water uptake at elevated RHs. The water sorption isotherm of human SC is typically categorized as type II based on the Brunauer et al. classification system^33,34^. However, because the isotherms in this study were obtained in relatively large RH steps, particularly in the 0–20% RH range, the characteristic sigmoidal shape of these isotherms is not clearly observed, making it difficult to distinguish between type II and III^33^. Notably, the SC samples pretreated with NIA show similar water sorption behavior compared to untreated SC up to 90% RH (Fig. 1A). However, at 95% RH, a significant increase in water uptake is observed for NIA-treated SC, despite NIA itself being non-hygroscopic, as indicated by its negligible water uptake in powder form (Fig. 1A). This finding suggests that the increased SC hydration cannot be attributed to NIA’s inherent ability to attract water. The elevated SC water content at 95% RH following NIA treatment was consistent across all four buffer types, with significant increases in three out of four cases (Fig. 1C, Table S1). At 60% RH, the SC water content is minimally affected by different treatments, though NIA-treated samples show a slight but consistent reduction in SC water content (about 1 wt%). This effect is statistically significant in one case (Fig. 1B, Table S1). SC samples soaked in saline buffers exhibit a consistent increase in water content at both RH levels compared to corresponding buffers without extra salt, an effect that is significant in all cases at 95% RH (Table S2). However, the pH of the buffer (acidic vs. neutral) has negligible impact on SC hydration, with only one out of eight cases showing a significant difference (Table S3).

**Fig. 2:**
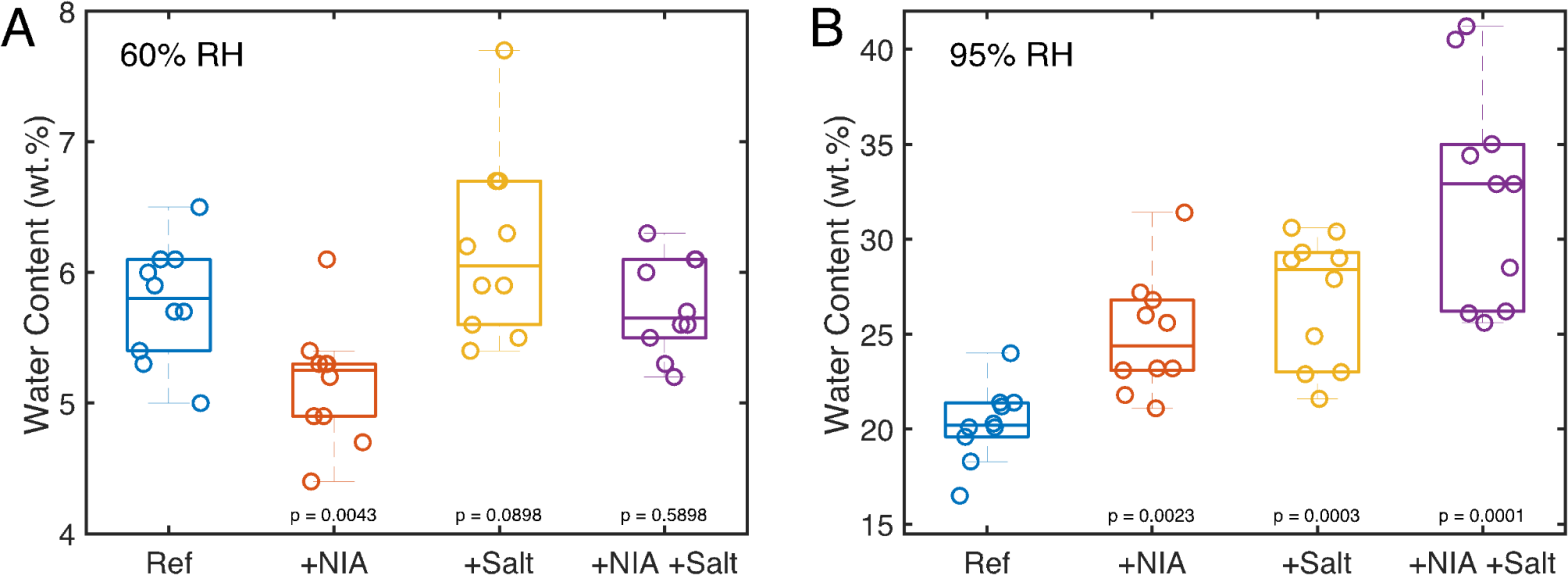
The effect of pretreatment condition on the SC water content at 60% RH (**A**) and 95% RH (**B**). The data correspond to the data presented in Fig. 1, where Ref corresponds to CB or PB (*n =* 10), +NIA corresponds to CB + NIA or PB + NIA (*n =* 10), +Salt corresponds to CBS or PBS (*n =* 10), +NIA + Salt corresponds to CBS + NIA or PBS + NIA (*n =* 10).

In summary, the results indicate that soaking the SC samples in different aqueous solutions affects their water content differently at 60% and 95% RH. At 60% RH, treatment with NIA alone (without additional salt) slightly reduces the water content, whereas at 95% RH, it increases the water content compared to samples treated without NIA. In contrast, treatments with additional salt (without NIA) increase the SC water content at both humidity levels. When treated with both NIA and extra salt, the effects counterbalance at 60% RH, maintaining baseline water content, but act synergistically at 95% RH, leading to higher water content. Figure 2 provides a summary of these results, showing the SC water content under various pretreatment protocols at both RH levels.

### Hydration of stratum corneum expands the interchain separation of keratin monomers and enhances the X-ray diffraction profile from the lipid lamellar matrix

To investigate NIA’s impact on the molecular structure of SC proteins and the SC lipid matrix, we conducted X-ray diffraction measurements on SC samples following the same treatment protocols as in the DVS studies. To provide a general context for the SAXD and WAXD data, it is useful to briefly outline the structural information obtained by X-ray diffraction when employed on SC samples. Due to that the majority of CERs and FFAs of the SC lipids are saturated and unusually long, the lipid matrix represents a well-ordered and solid multilamellar structure^2–4,35–39^. X-ray diffraction is therefore an effective method for examining its spatial organization, which has yielded significant insights into the structure of the SC^35,36,38–40^. Specifically, SAXD provides information on the thickness of the repeating multilamellar lipid units. The lamellar structures of the SC lipid matrix are commonly discussed in terms of a long-period lamellar phase (about 11–13 nm) and a short-period lamellar phase (about 6 nm)^35,36,39^. However, it has also been proposed that the complex diffraction pattern of the SC lipids can be attributed to a single phase comprised of a low number of repeating units (e.g., 2–3) of the long periodicity phase^36^. The WAXD data, in contrast, reveal short-range ordering, including the lateral packing of the solid lipid chains (i.e., orthogonal to the lamellar structures). The lateral organization of the lipid hydrocarbon chains normally coexist in the forms of orthorhombic (spacings about 0.37 nm and 0.41 nm) and hexagonal (spacing about 0.41 nm) unit cells in human SC^5,35^. For example, the presence of domains with different lateral packing within the SC lipid matrix has been observed, with orthorhombic packing being more abundant in the middle layers and hexagonal packing dominating the top and bottom surface layers^40^. These findings suggest that the SC lipid matrix has a complex organization, which may comprise sublayers with varying lamellar repeat distances and also phase-separated domains with varying lateral packing of the lipid hydrocarbon chains. Additionally, the WAXD data can identify features of soft keratin in the SC^35^. In particular, a broad peak corresponding to about 1 nm spacing reflects the distance between α-helix chains within the rigid core of keratin filaments^34,41^. Together, the SAXD and WAXD data provide insights into the hierarchical organization of the SC, including both lipid and keratin structures, enabling a comprehensive understanding of its structural properties.

**Fig. 3:**
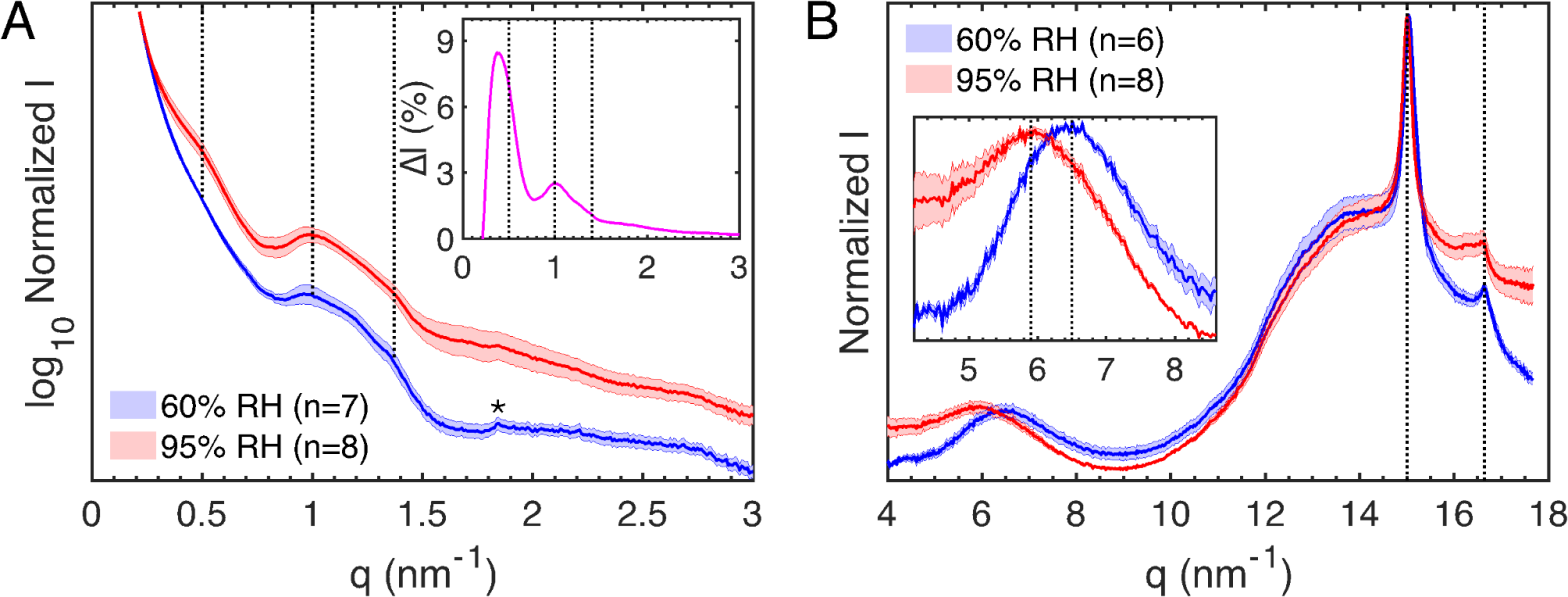
SAXD (**A**) and WAXD (**B**) diffraction data of untreated SC sheets equilibrated at 60% RH (blue) and 95% RH (red). Insert in (A) show the difference in normalized intensity between the diffraction curves obtained at 95% RH and 60% RH (i.e., $\:\varDelta\:\varvec{I}={\varvec{I}}_{\varvec{n}}^{95\varvec{R}\varvec{H}}-{\varvec{I}}_{\varvec{n}}^{60\varvec{R}\varvec{H}}$). Dotted lines in (**A**) indicate the expected periodic spacing of a 13 nm lamellar phase^36^, while the asterisk indicates phase-separated domains of solid cholesterol^39^. Insert in (B) is a close up of the broad diffraction peak from soft keratin with dotted lines indicating the keratin monomer spacings. The dotted lines at *q =* 15.0 nm^−1^ and *q =* 16.6 nm^−1^ denote peaks originating from solid lipids with hexagonal and/or orthorhombic lateral packing^5,35^. The water contents, based on DVS measurements, were 6.0 ± 0.1 wt% and 23.6 ± 1.0 for the SC samples equilibrated at 60% and 95% RH, respectively.

To separate NIA’s specific effects from general hydration effects, we initially focused on samples pretreated in buffers without NIA, equilibrated at either 60% or 95% RH. Due to biological variation and the inherent complexity of X-ray diffraction data from bulk SC samples, individual diffraction profiles showed notable variability, making it challenging to detect distinct patterns (Figs. S2, S3). This is not surprising given the well-known variability of X-ray diffraction data on isolated SC sheets^5,36,38,40,42^. Therefore, we averaged the normalized intensity values from these diffraction curves at each RH level, regardless of buffer type. The resulting averaged SAXD and WAXD profiles, shown in Fig. 3A,B, respectively, reveal clearer trends.

The SAXD data in Fig. 3A show broad peaks, or shoulders, at *q*-values centered at approximately 0.5, 1, and 1.4 nm⁻¹. This aligns well with a recent microbeam X-ray diffraction study on human SC, indicating that the lipid matrix is organized in at least one lamellar phase with repeat distance of approximately 13 nm ($\:{d}_{n}=2\pi\:/{q}_{n}$ where $\:n$ is the order of the peak)^36^. Notably, the shoulder centered around 0.5 nm^−1^ becomes more pronounced for the more hydrated SC samples, equilibrated at 95% RH. This is clearly shown in the insert in Fig. 3A, which shows the intensity difference between the diffraction curves obtained at 95% RH and 60% RH. Additionally, the weak diffraction peak at 1.84 nm^− 1^, attributed to phase-separated cholesterol^39^, is more defined in the samples equilibrated at 60% RH. Overall, these observations indicate that hydration affects the diffraction profiles originating from the lipid matrix, although without any detectable swelling of the lamellar unit cell^39^. For instance, the incorporation of a few water molecules into the headgroup regions of the lipid lamellae may slightly increase the electron density, resulting in stronger diffraction profiles, without any swelling of the lamellar repeat distance^39^. In addition, hydration of the SC also leads to increased lipid mobility^37^, which may influence lipid mixing and thereby explain why the diffraction from phase-separated cholesterol is reduced^42^.

Turning to the WAXD data in Fig. 3B, the most prominent peak is located around *q =* 15.0 nm^−1^ (*d =* 0.42 nm), while a weaker diffraction peak is observed at *q =* 16.6 nm^−1^ (*d =* 0.38 nm). These diffraction peaks are indicative of solid lipid phases that are laterally organized in hexagonal and/or orthorhombic unit cells. Specifically, the orthorhombic unit cell produces diffraction peaks at approximately *q* = 15 nm^−1^ and *q* = 17 nm⁻¹, whereas the hexagonal unit cell is associated with a single peak around *q* = 15 nm⁻¹^5,35^. Consequently, the results suggest that the SC lipids are arranged in a combination of hexagonal and orthorhombic unit cells. This coexistence implies domain formation, or even phase separation, within the solid lipid lamellar matrix. However, since the peak around 15 nm^−1^ may originate from both hexagonally and orthorhombically arranged lipids, it is challenging to accurately determine the proportions of these different domains/solid phases. The second peak originating from the orthorhombic unit cell at *q =* 16.6 nm^−1^ is more distinct at 60% RH. However, an analysis of the individual diffraction profiles at 95% RH shows that some of these samples are associated with a slight peak shift to 16.2 nm^−1^ (Fig. S3), implying that the averaged peak become less defined due to sample variability.

The broad diffraction peaks centered around *q =* 6.46 ± 0.03 nm^−1^ (*d =* 0.97 ± 0.01 nm) and *q =* 5.94 ± 0.07 nm^−1^ (*d =* 1.06 ± 0.01 nm) for the samples equilibrated at 60% and 95% RH, respectively, are attributed to the distance between adjacent keratin monomers^34,41^. As clearly shown in the insert in Fig. 3B, this peak is shifted towards lower *q*-values due to water uptake, which correspond to swelling of the keratin filaments of about 0.7-1.0 Å. This finding is in good agreement with previous X-ray diffraction studies investigating the effect of hydration of human SC^34,41^.

The results in Fig. 3 highlight the impact of hydration on the SC’s molecular structure. Considering this, in combination with the water sorption measurements showing that SC samples treated in saline buffers absorb more water, especially at 95% RH, we performed additional comparisons. SAXD profiles reveal that saline pretreatment enhances lipid peak intensity at 95% RH but has minimal effect on keratin swelling at either 60% RH or 95% RH (Fig. S4). Comparisons between acidic and neutral buffers indicate slightly increased lipid diffraction at pH 7.4, with no impact on keratin swelling (Fig. S5). Overall, these findings suggest that the SC water content primarily influences its molecular organization, while salt and, to a lesser extent, pH may affect water uptake and thus indirectly influence the SC structure. Following this hydration assessment, we aim to examine NIA’s effects on SC lipid and keratin organization at 60% and 95% RH.

### Effects of niacinamide on the molecular organization of the stratum corneum

The X-ray diffraction results from SC samples pretreated in buffers containing 5 wt% NIA and finally equilibrated at either 60% RH or 95% RH are presented for each individual sample in Fig. S6 (SAXD) and Fig. S7 (WAXD). Again, considering the noticeable biological variation between samples, it is difficult to identify clear effects due to NIA pretreatment with either acidic or neutral buffers, with or without salt. However, one clear observation is that the SC samples pretreated in neutral buffers and finally equilibrated at 60% RH show significantly more phase-separated crystalline NIA in the WAXD profiles (Fig. S7). To highlight this finding, we present the results separately for acidic buffers and neutral buffers with 5 wt% NIA, along with comparisons to the corresponding buffers without NIA.

**Fig. 4:**
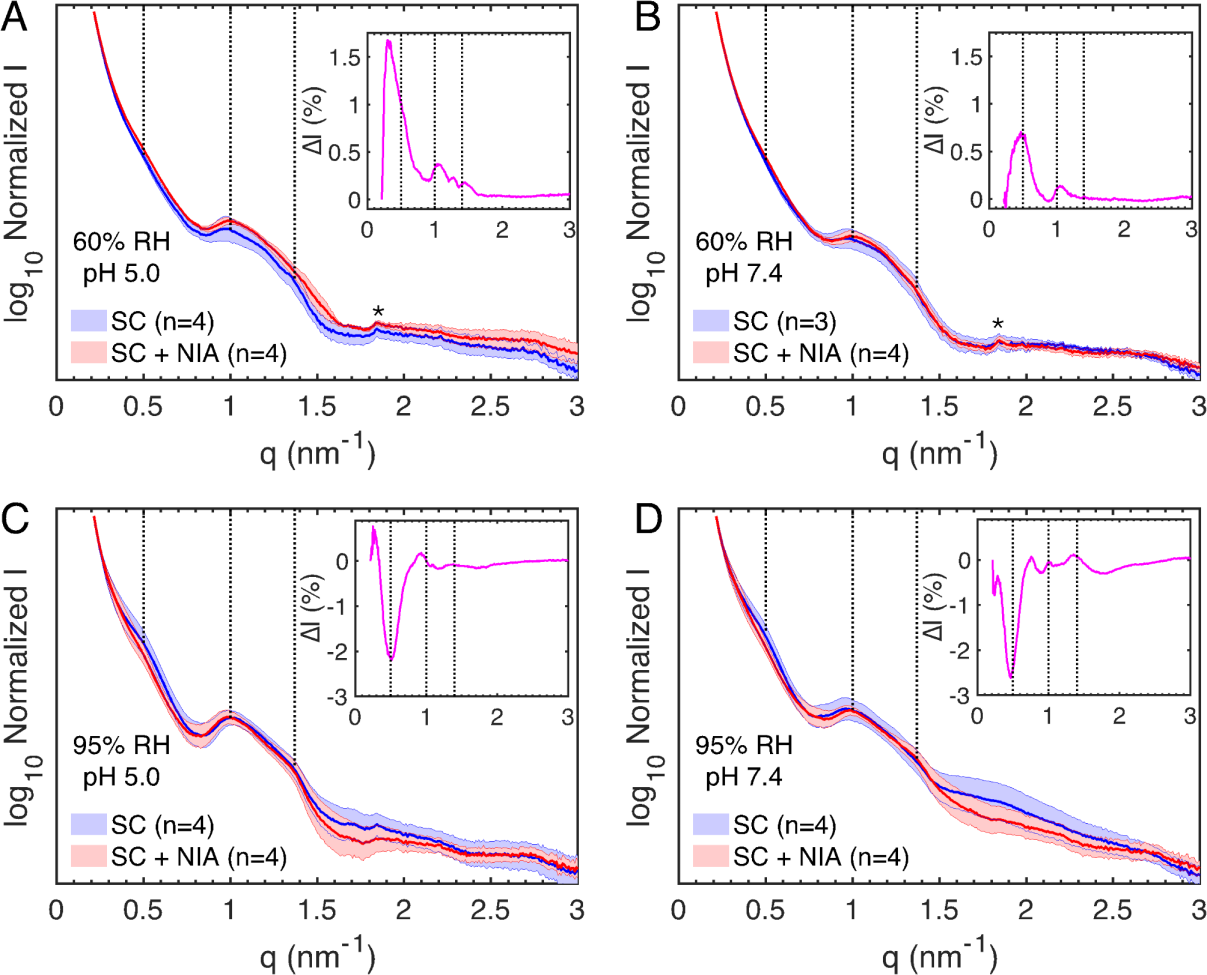
SAXD data from untreated SC (blue curves) and SC sheets treated in different buffers containing 5 wt% NIA (SC + NIA, red curves) and subsequently equilibrated in 60% RH (**A**,**B**) or 95% RH (**C**,**D**). The effect of NIA was investigated both in acidic (pH 5.0 in **A ** and **C**) and neutral conditions (pH 7.4 in **B** and **D**). Inserts show the difference in normalized intensity between the diffraction curves obtained from treated and untreated SC samples (i.e., $\:\varDelta\:\varvec{I}={\varvec{I}}_{\varvec{n}}^{\varvec{S}\varvec{C}+\varvec{N}\varvec{I}\varvec{A}}-{\varvec{I}}_{\varvec{n}}^{\varvec{S}\varvec{C}}$). Dotted lines indicate the expected periodic spacing of a 13 nm lamellar phase^36^, while the asterisks in (A and B) indicate phase-separated domains of solid cholesterol^39^.

### Niacinamide affects the SAXD intensity profiles oppositely at 60% and 95% relative humidity

Starting with the SAXD data (Fig. 4), which primarily reflect diffraction from the SC lipid matrix, the results obtained after equilibration at 60% RH are overall similar (Fig. 4A,B). One exception is that the samples pretreated with NIA show slightly elevated intensity from the main diffraction originating from the first-order lipid lamellar peak, which is highlighted in the inserts. In contrast, the samples equilibrated at 95% RH show the opposite trend, with reduced intensity from the main diffraction peak from SC lipids (Fig. 4A,B). Notably, these relative changes of the diffraction intensity profiles, occurring oppositely at 60% and 95% RH, are consistent in the case of both acidic (Fig. 4A,C) and neutral buffers (Fig. 4B,D).

According to the results presented in Fig. 3, elevated SC water content is expected to increase the diffraction intensity from the lipid matrix. Thus, these observations are surprising considering that the NIA-treated SC samples showed consistently lower water contents at 60% RH (Figs. 1B and 2A) and consistently higher water contents at 95% RH (Figs. 1C and 2B). Although the mechanism behind these findings remains unclear, it indicates that NIA either interacts directly with the SC lipid matrix or affects the distribution of water molecules in the lipid and protein compartments of the SC. For example, NIA might influence the partitioning of water molecules in the headgroup regions of the lipid lamellae, effectively changing the expected electron density profiles occurring in these regions. In turn, this mechanism may depend on how NIA is distributed in the SC. As a first approximation, NIA is expected to be preferentially distributed in the more hydrophilic domains of the SC, such as the corneocytes, with limited distribution in the lipid matrix. This leads us to the next section, where we analyze the WAXD data to examine the organization of the keratin filaments, as well as the lateral packing of the solid lipid matrix.

### Niacinamide mimics the effect of water in dry conditions leading to swelling of the interchain distance of keratin monomers

**Fig. 5:**
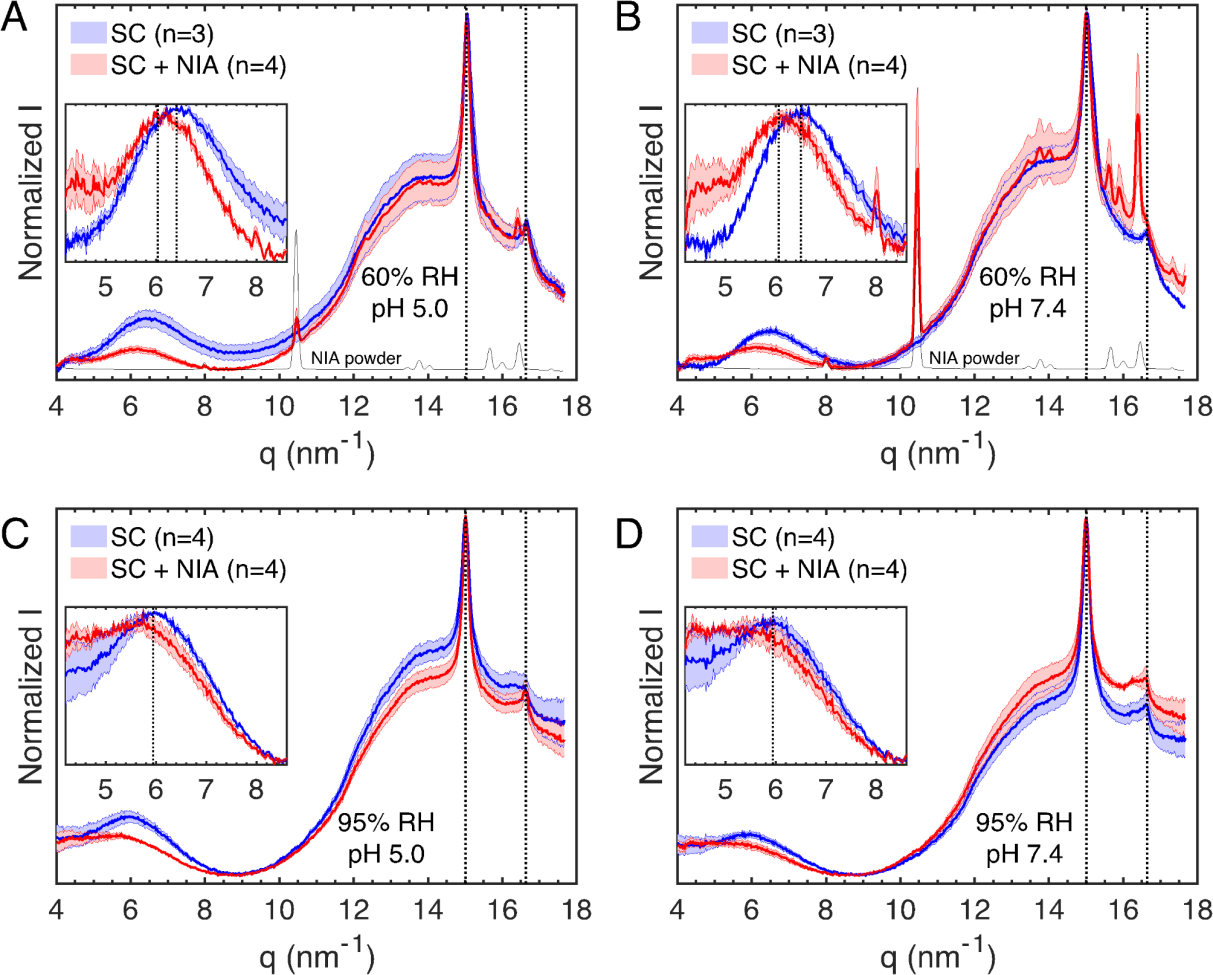
WAXD data from untreated SC sheets (blue curves) and SC samples treated in different buffers containing 5 wt% NIA (SC + NIA, red curves) and subsequently equilibrated at 60% RH (**A**,**B**) or 95% RH (**C**,**D**). The effect of NIA was investigated both in acidic (pH 5.0 in **A** and **C**) and neutral conditions (pH 7.4 in **B** and **D**). Inserts are close ups of the broad diffraction peak from soft keratin with dotted lines indicating the keratin monomer spacings^34,41^. The dotted lines at *q =* 15.0 nm^−1^ and *q =* 16.6 nm^−1^ denote peaks originating from solid lipids with hexagonal and/or orthorhombic lateral packing^5,35^. For comparison, the diffraction profile from pure NIA in powder form is included in (**A**) and (**B**), plotted with arbitrary intensity values.

Figure 5 shows WAXD data for SC samples treated with 5 wt% NIA and equilibrated at 60% RH or 95% RH. As mentioned above, the results are divided by buffer pH (5.0 and 7.4) to highlight the more pronounced precipitation of crystalline NIA in the case of neutral pH. Still, in both cases, strong diffraction peaks are observed at *q*-values overlapping with the diffraction peaks from pure NIA in powder form. This indicates that NIA penetrates the SC tissue readily during the pretreatment step. As a result, the SC samples become oversaturated with NIA after the water content is reduced by equilibration at 60% RH, leading to the formation of crystalline NIA depots. Thus, the results imply that the penetration of NIA into the SC tissue is elevated at pH 7.4 compared to pH 5.0. Except for this difference, the overall diffraction profiles for the NIA-treated SC samples are similar. A caveat of this conclusion is that the second peak from lipids with an orthorhombic lateral packing (*q =* 16.6 nm^− 1^) is almost overlapping with one of the NIA peaks. This is particularly problematic for the SC samples treated with NIA in PBS, which is associated with prominent diffraction peaks from crystalline NIA (Fig. 5B), making it difficult to draw any conclusions regarding the effect of NIA on the orthorhombic lipid packing in this case.

A comparison of the NIA-treated samples with untreated samples reveals a clear effect on the broad peak from soft keratin, which becomes weaker and shifted to lower *q*-values due to NIA. As highlighted in the inserts of Fig. 5A,B, in the case without NIA, the broad keratin peaks are centered around *q* = 6.41 ± 0.06 nm^−1^ (*d* = 0.98 ± 0.01 nm) for pH 5.0 and *q* = 6.48 ± 0.02 nm^−1^ (*d* = 0.97 ± 0.00 nm) for pH 7.4. In contrast, following NIA treatment, these peaks are shifted to lower *q*-values of around 6.03 ± 0.08 nm^−1^ (*d* = 1.04 ± 0.01 nm) and *q* = 6.06 ± 0.17 nm^−1^ (*d* = 1.04 ± 0.03 nm), respectively. Thus, this effect is observed irrespective of pH and corresponds to an expansion of the keratin monomer separation of about 0.4-1.0 Å. Notably, this resembles the swelling effect due to SC hydration, which was around 0.7-1.0 Å. However, since the NIA-treated SC samples consistently have lower water contents at this RH level, this effect is uniquely attributed to the presence of NIA and not to hydration-induced swelling. In conclusion, these observations indicate that NIA primarily partitions into the corneocytes where it expands the monomer separation of keratin filaments at 60% RH in a similar manner as incorporation of water molecules at elevated humidities.

The overall weaker diffraction from soft keratin suggests that NIA increases keratin disorder, possibly even leading to partial disentanglement. This observation led to the hypothesis that NIA might act as a keratolytic agent, similar to urea, a substance often used to exfoliate highly keratinized SC. To test this, we conducted additional experiments on SC samples pretreated with varying NIA concentrations, with the aim of observing progressively weaker keratin diffraction. SC samples were treated with PBS containing 2.5, 5.0, 10, or 20 wt% NIA for 24 h, then equilibrated at 60% RH. However, no dose-dependent reduction in keratin diffraction was found, although a trend of increased crystalline NIA with higher concentrations was observed (Fig. S8). These findings suggest that NIA does not disintegrate soft keratin structures and operates through a different mechanism than a keratolytic agent.

Figure 5C and D show WAXD data at 95% RH, revealing two key effects. First, phase-separated crystalline NIA is no longer present at either pH 5.0 or pH 7.4, suggesting that hydration leads to the dissolution of NIA depots within the SC tissue. Second, following NIA-treatment, the diffraction from soft keratin is relatively weak and possibly shifted to even lower *q*-values as compared to the untreated SC samples, which are associated with more defined keratin peaks centered around 5.94 ± 0.07 nm^−1^ (*d* = 1.06 ± 0.01 nm) as indicated by the dotted lines in the inserts of Fig. 5C,D. However, considering the undefined shape of the keratin diffraction peaks following NIA-treatment and equilibration at 95% RH, it is difficult to identify the precise peak position in these cases (see Fig. 5C,D). Given that NIA-treated SC contains about 5 wt% more water, along with dissolved NIA, these findings likely result from the combined effects of NIA and increased hydration at 95% RH. Finally, the diffraction peaks from solid lipids laterally arranged in orthorhombic and/or hexagonal unit cells are overall similar when comparing the NIA-treated SC samples with the untreated SC samples at 95% RH.

### The observed effects of niacinamide are not dependent on the presence of extra salt or on the pH of the treatment media

To confirm that the observed effects of NIA were not influenced by the salinity or pH of the pretreatment media, we conducted further comparisons of the SAXD and WAXD data at both 60% and 95% RH. Briefly, the results for treatments in saline buffers with NIA closely resemble those in non-saline buffers with NIA, with minor differences attributed to the increased SC water content after pretreatment with saline buffers (Fig. S9). Similarly, comparisons between acidic and neutral buffers containing NIA show consistent results (Fig. S10). Overall, these findings suggest that NIA has specific effects on SC molecular organization, leading to expanded keratin monomer spacing and, to some extent, increased disorder of the soft keratin.

## Discussion

NIA is widely used in topical skincare products due to its satisfactory safety profile and diverse beneficial properties on the biological function of the skin^19–28^. However, biophysical studies on the direct effect of NIA on the SC hydration process and the molecular organization of the lipid matrix and soft keratin structures of the SC are missing. To address this, the main aim of this study was to identify specific effects of NIA on the structural organization of the SC, as well as NIA’s effects on the SC hydration process. This was achieved by investigating SC samples pretreated in buffers containing 5 wt% NIA with different pH values and degrees of salinity. Reference samples were also included, pretreated in the corresponding buffers without NIA. Using this study design, we identified specific effects of NIA that cannot be attributed to variations in buffer pH or salinity. The most important findings are discussed below.

The mechanism of action of humectants is typically described as their ability to attract and retain water in the SC tissue [58]. However, on a molecular scale, several studies have shown that the presence of this class of molecules in the SC can maintain the molecular mobility of both lipid and protein segments in dry conditions with reduced SC water content^17,43,44^. Notably, this effect appears to be identical to how water affects the SC molecular mobility without added humectants^37^. An analogy can be made with biopolymer materials, where small polar molecules are used as plasticizers to lower the glass transition temperature and viscosity, enhancing their flexibility and softness in the dry state^45^. Considering that the SC often encounters a dry environment with low RH, which may lead to dry skin with diminished barrier function, it is plausible that incorporation of small polar molecules, such as NIA, can maintain SC’s flexibility, softness, and pliability in a similar manner as water^12,13^.

With this background, the X-ray diffraction measurements on SC sheets, pretreated without NIA and equilibrated at 60% or 95% RH, reveal clear effects of the hydration process. Specifically, comparing less hydrated SC samples (60% RH) with more hydrated samples (95% RH) shows a distinct hydration-induced swelling of the keratin monomer spacings by approximately 0.7-1.0 Å (Fig. 3B). This finding is in line with previous studies on human SC showing a swelling of approximately 0.5–1.3 Å^34,41^. To explain this effect, we start by considering the structure of keratin filaments. The keratin fibrils consists of monomers with a rigid rod core with protruding random coils of the terminal N and C domains^15,16^. The monomers form polar dimers, which then create zigzagged and antiparallel tetramers that align both lengthwise and side-by-side to form apolar protofilaments, measuring 2–3 nm in diameter. The protofilaments assemble into protofibrils, which then assemble into the final keratin filament. This filament has a coiled-coil structure, consisting of about 16–32 polypeptides in cross-section, with a diameter of around 10 nm^17^. The broad diffraction peak from keratin represents the distance between the rigid cores of adjacent keratin monomer chains^34,41^, including both the thickness of the protein chain as well as the thickness of any sorbed water layer. Considering that the shift in separation between adjacent monomer chains is evidently smaller than the diameter of a water molecule (i.e., 2.8 Å), it is unlikely that the expansion is due to formation of an additional water layer. Instead, it is more likely that the swelling decrease the overall packing density of the keratin filaments, allowing the protruding terminal polypeptide chains to adopt more dynamic configurations. This suggestion aligns well with previous solid-state NMR studies on SC samples. These studies show that the mobility of specific amino acid residues, abundant in the terminal random coils of keratin monomers, increases above approximately 80% RH^37,44^. Conversely, at lower RH values such as 60% RH, the amino acid residues of the terminal domains remain completely rigid^23, 57]^. Thus, the hydration-induced swelling of soft keratin can explain the well-established effect of hydration, leading to more pliable and soft SC tissue, primarily due to water uptake in the keratin-enriched corneocytes^2,12,13,15^.

A similar effect is observed for the NIA-treated SC samples, resulting in increased spacing between the keratin monomers on the order of 0.4–1.0 Å (Fig. 5A and B). Notably, in the case of NIA treatment, this effect occurs at dry conditions when the SC water content is very low (i.e., around 5–6 wt%). In fact, the water sorption experiments at 60% RH show that pretreatment with NIA leads to slightly lower SC water contents compared to pretreatment in the corresponding buffers without NIA (Figs. 1B and 2A). In other words, this effect appears to specifically arise from the presence of NIA in the dry SC tissue, indicating that NIA molecules interact with the keratin filaments similarly to water molecules, thereby effectively increasing their interchain spacings. This effect can be expected to increase the overall flexibility and softness of keratin, resulting in an overall more pliable SC tissue. This mechanism of action is particularly relevant in dry conditions, where any addition of water via topical skin care will quickly evaporate while NIA stays behind to exert its effect. A similar swelling effect of the keratin filaments at 60% RH is not observed when comparing the X-ray diffraction results from SC samples pretreated in media with or without extra salt (Fig. S4), or in the case of pretreatment in acidic or neutral buffers (Fig. S5). This supports the conclusion that NIA specifically affects the soft keratin structures of the SC.

The sorption isotherms (Fig. 1A) show that NIA powder is non-hygroscopic, absorbing minimal water even at 95% RH (around 1 wt%). However, after pretreating with NIA and equilibrating the SC at 95% RH, there is a significant increase in water content compared to pretreatment without NIA (Figs. 1C and 2B). This observation is consistent across all buffers used as pretreatment media (Fig. 1C). Additionally, the results indicate that both NIA and NaCl have similar effects at 95% RH and act synergistically when combined, leading to significantly higher SC water content compared to pretreatment without NIA and extra salt (Fig. 2B). However, it is important to note that the biophysical properties of the SC, including the molecular mobility of its lipid and protein components, are not solely dependent on water content when non-volatile small polar molecules are present^17,31^. For example, a previous study focusing on the effects of glucose and NaCl on the water sorption behavior and the molecular mobility of SC showed that mixtures of these substances increase the molecular mobility of SC lipids and proteins more than the individual compounds^31^. Still, the SC sample containing only NaCl resulted in the highest water content at 95% RH^31^. With respect to this study, a similar conclusion can be made. As outlined above, the swelling effect of the keratin filaments at 60% RH is observed only with NIA treatment and not by treatment with extra salt (without NIA). This highlights the importance of not only focusing on the SC hydration degree but instead consider the overall effects from both water and the substance under investigation. In the case of application of topical skin care products aiming to alleviate dry and defective skin, it is well-known that the initial formulation undergoes metamorphosis^46^. In other words, volatile components like water evaporate quickly, while non-volatile substances such as NIA remain on the skin surface, where they can diffuse into and integrate with the SC tissue over time. Effectively, its presence inside the SC can maintain the molecular mobility of keratin and lipids in dry conditions, thereby improving the flexibility and barrier integrity of the SC, even with low water content. This in turn may improve the water uptake resulting from the continuous hydration process of the SC from within the body, driven by the water gradient between the water-rich tissue and the surrounding environment^12,47^. It is, of course, uncertain how the observed effects of NIA on the molecular organization of the SC translate into subsequent effects on biochemical and enzymatic processes that are important for maintaining a healthy SC barrier. However, the role of having adequate water levels of the SC for maintaining function of biochemical and enzymatic processes have been shown previously^47–49^, suggesting that molecular mobility of the SC matrix affects enzyme function. One may therefore speculate that keratin swelling induced by NIA may help to maintain molecular mobility within the SC matrix, which may facilitate optimal SC enzyme function in vivo when the skin is normally exposed to desiccating conditions.

The SAXD results show that increased SC hydration generally intensifies the diffraction profiles from the lipid matrix (see insert in Fig. 3A). However, NIA modifies this effect. At 95% RH, despite higher water content, NIA-treated samples show reduced diffraction intensity (see inserts in Fig. 4B,D), while at 60% RH, they exhibit increased intensity despite slightly lower water content (see inserts in Fig. 4A,C). These opposite effects at different RH levels are consistent across both acidic and neutral buffers. To rationalize these observations, we begin by estimating the NIA content in the SC tissue post-treatment. This estimation assumes that NIA molecules in the SC do not absorb water at 60% RH, resulting solely in a dilution of the SC sample’s dry weight. This assumption is supported by the fact that NIA powder does not absorb water at 60% RH, and NIA-treated SC samples consistently absorb less water at 60% RH compared to untreated samples, indicating a dilution of the SC dry weight. Based on this reasoning, the NIA content ($\:{C}_{Nia}$, wt%) is estimated using following equation:

1 $$\:{C}_{Nia}={C}_{SC,NIA}-{C}_{w,NIA}\times\:{C}_{SC}/{C}_{w}$$

In Eq. (1), $\:{C}_{SC,NIA}$ and $\:{C}_{w,NIA}$ represent the dry content and water content of the NIA-treated samples, respectively, while $\:{C}_{SC}$ and $\:{C}_{w}$ are the corresponding values for the untreated samples (all values at 60% RH and units in wt%). This calculation yields an average NIA content of approximately 9 wt% in the SC samples (*n =* 20). Based on the X-ray data, it appears that NIA primarily partitions within corneocytes, where it effectively expands the spacing between keratin chains. This expansion may change the corneocyte shape, potentially pressuring the surrounding lipid matrix and possibly increasing the diffraction profile of the lipid lamellae (Fig. 4A and B). Similar effects have been proposed with hydration-induced corneocyte swelling^50^. Furthermore, NIA-induced keratin expansion likely occurs due to interactions between NIA and polar amino acid residues of the protruding terminal domains of keratin monomers. Presumably, NIA and water compete for intermolecular interactions with the same amino acid side-groups, which may limit the water uptake by these groups at 60% RH if they are occupied by NIA interactions. Consequently, water molecules may redistribute toward the lipid head group regions at 60% RH, enhancing its electron density profiles and thus increasing the diffraction intensity (Fig. 4A and B). It should, however, be pointed out that the diffraction peaks originating from crystalline NIA, observed at 60% RH, suggest that the SC is oversaturated with NIA. This makes it unclear how much NIA is freely interacting with keratin at 60% RH. Here, it can be noted that pretreatment with lower NIA concentration (i.e., 2.5 wt%) also resulted in diffraction peaks from crystalline NIA after equilibration at 60% RH (Fig. S8). On the other hand, after equilibration at 95% RH, no diffraction peaks from crystalline NIA are observed, implying that all NIA is incorporated into the hydrated SC. Furthermore, at 95% RH, the presence of NIA significantly increases the SC water uptake, which most likely occurs due to water absorption by the corneocytes, possibly driven by the osmotic pressure buildup due to dissolved NIA molecules. Considering that SC hydration leads to increased lipid mobility^37,42^, it is possible that the combined effect of NIA and elevated water content results in a less ordered lipid matrix. This could explain the reduced diffraction intensities compared to the untreated samples at 95% RH (Fig. 4C,D).

In summary, this study aimed at investigating the effects of NIA on the hydration process and molecular organization of the SC. By comparing SC samples treated with NIA under different humidity conditions to untreated controls, the study reveals distinct effects of NIA that are independent of buffer pH or salinity variations of the pretreatment media. Key conclusions from the study include:

- NIA is non-hygroscopic in its powdered form, but when incorporated into the SC, it enhances water uptake significantly at high relative humidity (95% RH). This effect is consistent across different buffer solutions used as pretreatment medium.
- At low humidity levels (60% RH), NIA swells the interchain distance between keratin monomers, mimicking the effect of water. This indicates that NIA may function as a plasticizer and potentially increase the flexibility and softness of the SC in dry conditions. Considering that NIA remains in the SC after water evaporates, it can exert its effects in desiccating environments, supporting its beneficial functions in skincare formulations aimed at improving skin barrier function.
- SAXD and WAXD data show that NIA alters the lipid diffraction patterns differently at 60% and 95% RH. Specifically, NIA increases lipid diffraction intensity at 60% RH while reducing it at 95% RH, indicating interactions with the lipid matrix and/or redistribution of water within the protein and lipid domains of the SC.
- Increased concentrations of NIA do not show a dose-response effect on the molecular structure of the SC. Even at higher concentrations in the pretreatment media, NIA induces similar effects on the keratin and lipid matrix, implying that a specific concentration threshold may be sufficient to achieve its effects without additional impact from higher doses. The results do not indicate that NIA acts as a traditional keratolytic agent. Instead, NIA seems to interact uniquely with keratin, resulting in expansion of keratin filaments without clear signs of their disintegration.

## Materials and methods

### Materials

Niacinamide, NaCl, KH_2_PO_4_, Na_2_HPO_4_·2H_2_O, LiCl, NaOH, HCl, and trypsin (from bovine pancreas) were obtained from Sigma-Aldrich. Phosphate buffered saline (PBS, pH 7.4) was prepared by mixing 130.9 mM NaCl, 5.1 mM Na_2_HPO_4_·2H_2_O, and 1.5 mM KH_2_PO_4_. Citrate buffered saline (CBS) was prepared by mixing 130.9 mM NaCl, 5.8 mM sodium citrate (C_6_H_7_NaO_7_), and 4.2 mM citric acid (C_6_H_8_O_7_). The corresponding buffers, without NaCl, were prepared in the same way, i.e., phosphate buffer (PB) and citrate buffer (CB). For all buffers, the pH was adjusted to obtain pH 7.4 (PB and PBS) and pH 5.0 (CB and CBS) by adding aliquots of 2 M NaOH or 1 M HCl. All aqueous solutions were prepared using Millipore water with 18.2 MΩ·cm.

## Stratum corneum preparation

Human skin was obtained from three healthy female donors aged 59, 38, and 55 years, who underwent plastic surgery and had previously signed written informed consent. The process was approved by the Swedish Ethics Committee (no. 2018/509 − 32) and conducted in accordance with the principles of the Declaration of Helsinki. The fat tissue was removed from the skin using a scalpel, and the SC was subsequently isolated through trypsin digestion^51^. For this procedure, the skin tissue was placed dermal side down on filter paper soaked with 0.1 wt% trypsin in PBS and refrigerated overnight. The following day, the SC was separated from the underlying tissue using tweezers, then carefully washed with PBS and cotton swabs to remove any residual tissue. Finally, it was dried inside a desiccator under reduced pressure.

Prior to X-ray and water sorption measurements, appropriate amounts of dry SC were placed in 2 ml of aqueous buffer solutions, with or without 5 wt% NIA. In total, four different buffer solutions were employed, which differed in pH value and salt concentration as follows: PB (phosphate buffer without extra NaCl, pH 7.4), PBS (phosphate buffered saline, pH 7.4), CB (citrate buffer without extra NaCl, pH 5.0), and CBS (citrate buffered saline, pH 5.0). After soaking the SC samples in buffer for 24 h, the SC pieces were removed from the solutions and dipped three times in water and finally gently wiped with a paper tissue to remove excess solution. For water sorption measurements, the SC samples were dried inside a desiccator with reduced pressure for a minimum of 24 h. For X-ray measurements, the SC samples were equilibrated in humidity chambers at room temperature with 60% RH or 95% RH for 3–7 days before loading the SC into the sample holders. Based on the present results, no significant differences in SC organization or water sorption properties could be attributed to age differences of the three donors, aside from the natural biological variation inherent in SC samples.

## Dynamic vapor sorption (DVS)

Water sorption isotherms were measured at 25 °C using a DVS microbalance (Q5000 SA, TA Instruments - Waters Sverige AB, Sollentuna, Sweden). Dried SC pieces (approximately 3–6 mg) were placed in a glass cup, which was loaded onto the DVS microbalance and sealed in the sample chamber, where the RH is precisely controlled. Initially, the sample was exposed to dry N_2_ gas (0% RH) at 35 °C until a stable reading was achieved. In all cases, a stable reading was defined by a mass change < 0.02%. Next, the temperature was changed to 25 °C (0% RH) until a stable dry weight reading was obtained. After this, the relative humidity (RH) was ramped from 0%, 20%, 40%, 60%, 80%, 90%, to 95%. The sample mass was continuously measured by the microbalance and the water content at each RH value was calculated using the following equation $\:{m}_{w}=\left({m}_{tot}-{m}_{dry}\right)/{m}_{tot}\times\:100\%$, where $\:{m}_{w}$ is the water content of the sample, $\:{m}_{tot}$ is the total mass of the sample, and $\:{m}_{dry}$ is the dry mass of the sample at 0% RH and 25 °C. Based on these measurements, additional replicates were performed at 60% RH and 95% RH to collect sufficient data for statistical analysis (*n =* 5 in total).

### Small and wide-angle X-ray diffraction (SAXD and WAXD)

X-ray diffraction measurements were performed with a Xeuss 3.0 instrument (Xenocs, France), using Cu Kα radiation with a wavelength (*λ*) of 1.542 Å. The instrument is equipped with a Pilatus3 R 300 K hybrid photon-counting detector, set at a sample-to-detector distance of 800 mm for SAXD measurements and 285 mm for WAXD measurements. These settings cover a *q*-range of 0.01–18 nm^−1^ , where *q* is the scattering vector defined as *q* =4π/*λ* sin(*θ*/2), with *θ* being the scattering angle. For measurements, the samples were mounted in a multi-purpose Peltier sample holder and sealed using Kapton films (DuPont™ Kapton^®^, 0.013 mm thickness, Goodfellow, England) and O-ring spacers, and then equilibrated at 32 °C. The samples were exposed for 3 h for SAXD and 1 h for WAXD. Azimuthal integration of the 2D patterns provided the 1D diffraction data, which were corrected for background scattering and normalized to the direct beam using Xenocs XSACT software (version 2.6). Silver behenate was used to calibrate the *q*-scale, and background subtraction of two empty Kapton films was performed.

The intensity data from SAXD and WAXD measurements were normalized between zero and unity by $\:{I}_{n}=\left({I}_{i}-{I}_{min}\right)/\left({I}_{max}-{I}_{min}\right)$ for a selected range of *q*-values. For SAXD, $\:{I}_{n}$ was calculated between *q =* 0.21–3.56 nm^−1^, while for WAXD, $\:{I}_{n}$ was calculated between *q =* 3.68–17.67 nm^−1^. For analyzing the spacings between adjacent polypeptide monomers of the soft keratin filaments, based on the WAXD data, $\:{I}_{n}$ was calculated between *q =* 4.17–8.60 nm^−1^. To estimate the *q*-position of the broad keratin peaks, a Gaussian model was fitted to the data, which enabled the identification of the peak location. To validate the accuracy of this method, an alternative approach was employed based on identifying the *q*-values corresponding to the five highest intensity values of the local maximum. Next, the average value of *q* was calculated. In both cases, data from individual SC samples were analyzed to enable calculation of the mean value of a dataset, with error estimation represented by the SEM. The outcomes of both methods were in overall good agreement. However, due to the relatively poor keratin diffraction for some samples (see Fig. S3 and Fig. S7), the data-driven approach (i.e., identification of the local five highest maxima) was judged to be more suitable and therefore used.

### Statistical analysis

The DVS data are presented as mean values, with error bars representing the standard error of the mean (mean ± SEM). For statistical analysis, these data were treated with independent one-sided (one-tailed) t-tests assuming equal variances. *p*-values of 0.05, 0.01, and 0.005 were considered statistically significant at 95% (*), 99% (**), and 99.5% (***) confidence levels, respectively. For boxplots, outliers were identified using the interquartile range (IQR = Q3 - Q1) method, where data points that fall below Q1-1.5×IQR or above Q3 + 1.5×IQR are defined as outliers. The X-ray data are presented as mean values of the normalized intensity (i.e., $\:{I}_{n}$) with shaded error bounds given by the standard error of the mean (i.e., mean ± SEM).

## Electronic supplementary material

Below is the link to the electronic supplementary material.

Supplementary Material 1

## Data Availability

Data supporting the findings of this study are available from the corresponding author on request.

## References

[1] Scheuplein, R. J. & Blank, I. H. Permeability of the skin. *Physiol. Rev.* **51**, 702–747 (1971). 4940637 10.1152/physrev.1971.51.4.702

[2] Iwai, I. et al. The human skin barrier is Organized as stacked bilayers of fully extended ceramides with Cholesterol Molecules Associated with the Ceramide Sphingoid Moiety. *J. Invest. Dermatol.* **132**, 2215–2225 (2012). 22534876 10.1038/jid.2012.43

[3] Sparr, E. et al. The stratum corneum barrier – from molecular scale to macroscopic properties. *Curr. Opin. Colloid Interface Sci.* **67** (2023).

[4] Bouwstra, J. A. et al. The skin barrier: an extraordinary interface with an exceptional lipid organization. *Prog Lipid Res.* **92**, 101252 (2023). 37666282 10.1016/j.plipres.2023.101252 PMC10841493

[5] Bouwstra, J. A., Gooris, G. S., Salomons-De Vries, M. A., van der Spek, J. A. & Bras, W. Structure of human stratum corneum as a function of temperature and hydration: a wide-angle X-ray diffraction study. *Int. J. Pharm.* **84**, 205–216 (1992).

[6] Sahle, F. F., Gebre-Mariam, T., Dobner, B., Wohlrab, J. & Neubert, R. H. H. Skin diseases Associated with the Depletion of Stratum Corneum Lipids and Stratum Corneum lipid substitution therapy. *Skin Pharmacol. Physiol.* **28**, 42–55 (2015). 25196193 10.1159/000360009

[7] Steinert, P. M. & Marekov, L. N. The proteins elafin, filaggrin, keratin intermediate filaments, loricrin, and small proline-rich protein-1 and protein-2 are isodipeptide cross-linked components of the human epidermal cornified cell-envelope. *J. Biol. Chem.* **270**, 17702–17711 (1995). 7543090 10.1074/jbc.270.30.17702

[8] Boiten, W., Helder, R., van Smeden, J. & Bouwstra, J. Selectivity in cornified envelop binding of ceramides in human skin and the role of LXR inactivation on ceramide binding. *Biochim. Biophys. Acta* **1864**, 1206–1213 (2019). 10.1016/j.bbalip.2019.05.003 31112754

[9] Swartzendruber, D. C., Wertz, P. W., Madison, K. C. & Downing, D. T. Evidence that the corneocyte has a chemically bound lipid envelope. *J. Invest. Dermatol.* **88**, 709–713 (1987). 3585054 10.1111/1523-1747.ep12470383

[10] Takeichi, T. et al. SDR9C7 catalyzes critical dehydrogenation of acylceramides for skin barrier formation. *J. Clin. Invest.* **130**, 890–903 (2020). 31671075 10.1172/JCI130675 PMC6994155

[11] Kreplak, L., Doucet, J., Dumas, P. & Briki, F. New aspects of the alpha-helix to beta-sheet transition in stretched hard alpha-keratin fibers. *Biophys. J.* **87**, 640–647 (2004). 15240497 10.1529/biophysj.103.036749 PMC1304386

[12] Blank, I. H. Further observations on factors which influence the water content of the stratum corneum. *J. Invest. Dermatol.* **21**, 259–271 (1953). 13096868 10.1038/jid.1953.100

[13] Blank, I. H. Factors which influence the water content of the stratum corneum. *J. Invest. Dermatol.* **18**, 433–440 (1952). 14938659 10.1038/jid.1952.52

[14] Norlén, L. Stratum corneum keratin structure, function and formation – a comprehensive review. *Int. J. Cosmet. Sci.* **28**, 397–425 (2006). 18489286 10.1111/j.1467-2494.2006.00345.x

[15] Bouwstra, J. A. et al. Water distribution and related morphology in human stratum corneum at different hydration levels. *J. Investig Dermatol.* **120**, 750–758 (2003). 12713576 10.1046/j.1523-1747.2003.12128.x

[16] Björklund, S., Engblom, J., Thuresson, K. & Sparr, E. Glycerol and urea can be used to increase skin permeability in reduced hydration conditions. *Eur. J. Pharm. Sci.* **50**, 638–645 (2013). 23643739 10.1016/j.ejps.2013.04.022

[17] Björklund, S. et al. Stratum corneum molecular mobility in the presence of natural moisturizers. *Soft Matter*. **10**, 4535–4546 (2014). 24817485 10.1039/c4sm00137k

[18] Björklund, S. et al. The effects of polar excipients transcutol and dexpanthenol on molecular mobility, permeability, and electrical impedance of the skin barrier. *J. Colloid Interface Sci.* **479**, 207–220 (2016). 27388135 10.1016/j.jcis.2016.06.054

[19] Marques, C. et al. *Mechanistic Insights into the Multiple Functions of Niacinamide: Therapeutic Implications and Cosmeceutical Applications in Functional Skincare Products, Antioxidants* 13 (2024). 10.3390/antiox13040425 PMC11047333 38671873

[20] Ong, R. R. & Goh, C. F. Niacinamide: a review on dermal delivery strategies and clinical evidence. *Drug Delivery Translational Res.* **14**, 3512–3548 (2024). 10.1007/s13346-024-01593-y 38722460

[21] Tanno, O., Ota, Y., Kitamura, N., Katsube, T. & Inoue, S. Nicotinamide increases biosynthesis of ceramides as well as other stratum corneum lipids to improve the epidermal permeability barrier. *Br. J. Dermatol.* **143**, 524–531 (2000). 10971324 10.1111/j.1365-2133.2000.03705.x

[22] Berson, D. S. et al. Niacinamide: A topical vitamin with wide-ranging skin appearance benefits. in *Cosmeceuticals and Cosmetic Practice* 103–112 ( 2013).

[23] Tan, C. Y. R. et al. Nicotinamide prevents UVB- and oxidative stress–Induced Photoaging in Human Primary keratinocytes. *J. Invest. Dermatol.* **142**, 1670–1681e1612 (2022). 34740582 10.1016/j.jid.2021.10.021

[24] Bierman, J. C. et al. Niacinamide mitigates SASP-related inflammation induced by environmental stressors in human epidermal keratinocytes and skin. *Int. J. Cosmet. Sci.* **42**, 501–511 (2020). 32657437 10.1111/ics.12651

[25] Tan, C. L. et al. Nicotinamide metabolism modulates the Proliferation/Differentiation balance and senescence of human primary keratinocytes. *J. Invest. Dermatol.* **139**, 1638–1647e1633 (2019). 30776433 10.1016/j.jid.2019.02.005

[26] Kimball, A. B. et al. Reduction in the appearance of facial hyperpigmentation after use of moisturizers with a combination of topical niacinamide and N-acetyl glucosamine: results of a randomized, double-blind, vehicle-controlled trial. *Br. J. Dermatol.* **162**, 435–441 (2010). 19845667 10.1111/j.1365-2133.2009.09477.x

[27] Bissett, D. L., Miyamoto, K., Sun, P., Li, J. & Berge, C. A. Topical niacinamide reduces yellowing, wrinkling, red blotchiness, and hyperpigmented spots in aging facial skin. *Int. J. Cosmet. Sci.* **26**, 231–238 (2004). 18492135 10.1111/j.1467-2494.2004.00228.x

[28] Rolfe, H. M. A review of nicotinamide: treatment of skin diseases and potential side effects. *J. Cosmet. Dermatol.* **13**, 324–328 (2014). 25399625 10.1111/jocd.12119

[29] Fukuda, K. et al. Three stepwise pH progressions in stratum corneum for homeostatic maintenance of the skin. *Nat. Commun.* **15**, 4062 (2024). 38750035 10.1038/s41467-024-48226-z PMC11096370

[30] Proksch, E. pH in nature, humans and skin. *J. Dermatol.* **45**, 1044–1052 (2018). 29863755 10.1111/1346-8138.14489

[31] Sonoki, Y., Dat Pham, Q., Sparr, E. & Additivity, B. A mixture of glucose and NaCl can influence skin hydration more than the individual compounds. *J. Colloid Interface Sci.* **613**, 554–562 (2022). 35065432 10.1016/j.jcis.2021.12.166

[32] Björklund, S. Skin hydration - How Water and Osmolytes Influence Biophysical Properties of Stratum Corneum. in *Physical Chemistry* (Lund University, 2013).

[33] Brunauer, S., Deming, L. S., Deming, W. E. & Teller, E. On a theory of the Van Der Waals Adsorption of gases. *J. Am. Chem. Soc.* **62**, 1723–1732 (1940).

[34] Hey, M. J., Taylor, D. J. & Derbyshire, W. Water sorption by human callus. *Biochim. Biophys. Acta*. **540**, 518–533 (1978).

[35] Hatta, I. Stratum Corneum structure and function studied by X-ray diffraction. *Dermato* **2**, 79–108 (2022).

[36] Yagi, N., Aoyama, K. & Ohta, N. Microbeam X-ray diffraction study of lipid structure in stratum corneum of human skin. *PLOS ONE*. **15**, e0233131 (2020). 32392265 10.1371/journal.pone.0233131 PMC7213682

[37] Björklund, S., Nowacka, A., Bouwstra, J. A., Sparr, E. & Topgaard, D. Characterization of stratum corneum molecular dynamics by natural-abundance 13 C solid-state NMR. *PLoS ONE*. **8**, e61889 (2013). 23626744 10.1371/journal.pone.0061889 PMC3633950

[38] Garson, J. C., Doucet, J., Leveque, J. L. & Tsoucaris, G. Oriented structure in human stratum corneum revealed by x-ray-diffraction. *J. Invest. Dermatol.* **96**, 43–49 (1991). 1987295 10.1111/1523-1747.ep12514716

[39] Bouwstra, J. A., Gooris, G. S., van der Spek, J. A. & Bras, W. Structural investigations of human stratum corneum by small-angle X-ray scattering. *J. Invest. Dermatol.* **97**, 1005–1012 (1991). 1748810 10.1111/1523-1747.ep12492217

[40] Doucet, J., Potter, A., Baltenneck, C. & Domanov, Y. A. Micron-scale assessment of molecular lipid organization in human stratum corneum using microprobe X-ray diffraction. *J. Lipid Res.* **55**, 2380–2388 (2014). 25180243 10.1194/jlr.M053389 PMC4617139

[41] Nakazawa, H., Ohta, N. & Hatta, I. A possible regulation mechanism of water content in human stratum corneum via intercellular lipid matrix. *Chem. Phys. Lipids*. **165**, 238–243 (2012). 22280854 10.1016/j.chemphyslip.2012.01.002

[42] Björklund, S. et al. Skin membrane electrical impedance properties under the influence of a varying water gradient. *Biophys. J.* **104**, 2639–2650 (2013). 23790372 10.1016/j.bpj.2013.05.008 PMC3686338

[43] Gunnarsson, M., Mojumdar, E. H., Topgaard, D. & Sparr, E. Extraction of natural moisturizing factor from the stratum corneum and its implication on skin molecular mobility. *J. Colloid Interface Sci.* **604**, 480–491 (2021). 34273783 10.1016/j.jcis.2021.07.012

[44] Mojumdar, E. H., Pham, Q. D., Topgaard, D. & Sparr, E. Skin hydration: interplay between molecular dynamics, structure and water uptake in the stratum corneum. *Sci. Rep.* **7**, 15712 (2017). 29146971 10.1038/s41598-017-15921-5 PMC5691061

[45] Vieira, M. G. A., da Silva, M. A., dos Santos, L. O. & Beppu, M. M. Natural-based plasticizers and biopolymer films: a review. *Eur. Polymer J.* **47**, 254–263 (2011).

[46] Surber, C. & Knie, U. Metamorphosis of vehicles: mechanisms and opportunities. *Curr. Probl. Dermatol.* **54**, 152–165 (2018). 30130783 10.1159/000489529

[47] Harding, C. R., Watkinson, A., Rawlings, A. V. & Scott, I. R. Dry skin, moisturization and corneodesmolysis. *Int. J. Cosmet. Sci.* **22**, 21–52 (2000). 18503460 10.1046/j.1467-2494.2000.00001.x

[48] Katagiri, C., Sato, J., Nomura, J. & Denda, M. Changes in environmental humidity affect the water-holding property of the stratum corneum and its free amino acid content, and the expression of filaggrin in the epidermis of hairless mice. *J. Dermatol. Sci.* **31**, 29–35 (2003). 12615361 10.1016/s0923-1811(02)00137-8

[49] Watkinson, A., Harding, C., Moore, A. & Coan, P. Water modulation of stratum corneum chymotryptic enzyme activity and desquamation. *Arch. Dermatol. Res.* **293**, 470–476 (2001). 11758790 10.1007/s004030100257

[50] Norlén, L., Axelsson, E. & Forslind, B. Stratum corneum swelling. Biophysical and computer assisted quantitative assessments. *Arch. Dermatol. Res.* **289**, 506–513 (1997). 9341970 10.1007/s004030050231

[51] Kligman, A. M. & Christophers, E. Preparation of isolated sheets of human stratum corneum. *Arch. Dermatol.* **88**, 702–705 (1963). 14071437 10.1001/archderm.1963.01590240026005

