# Supplementary material for: Niacinamide and its impact on stratum corneum hydration and structure
Source: Sci Rep. 2025 Feb 10;15:4953. doi: 10.1038/s41598-025-88899-0 (PMC11811021; doi:10.1038/s41598-025-88899-0)
Supplement: Supplementary file 1 — Supplementary Material 1 [file 41598_2025_88899_MOESM1_ESM.pdf]

Supportive Information
Niacinamide and Its Impact on Stratum Corneum Hydration and Structure

Supportive Figures

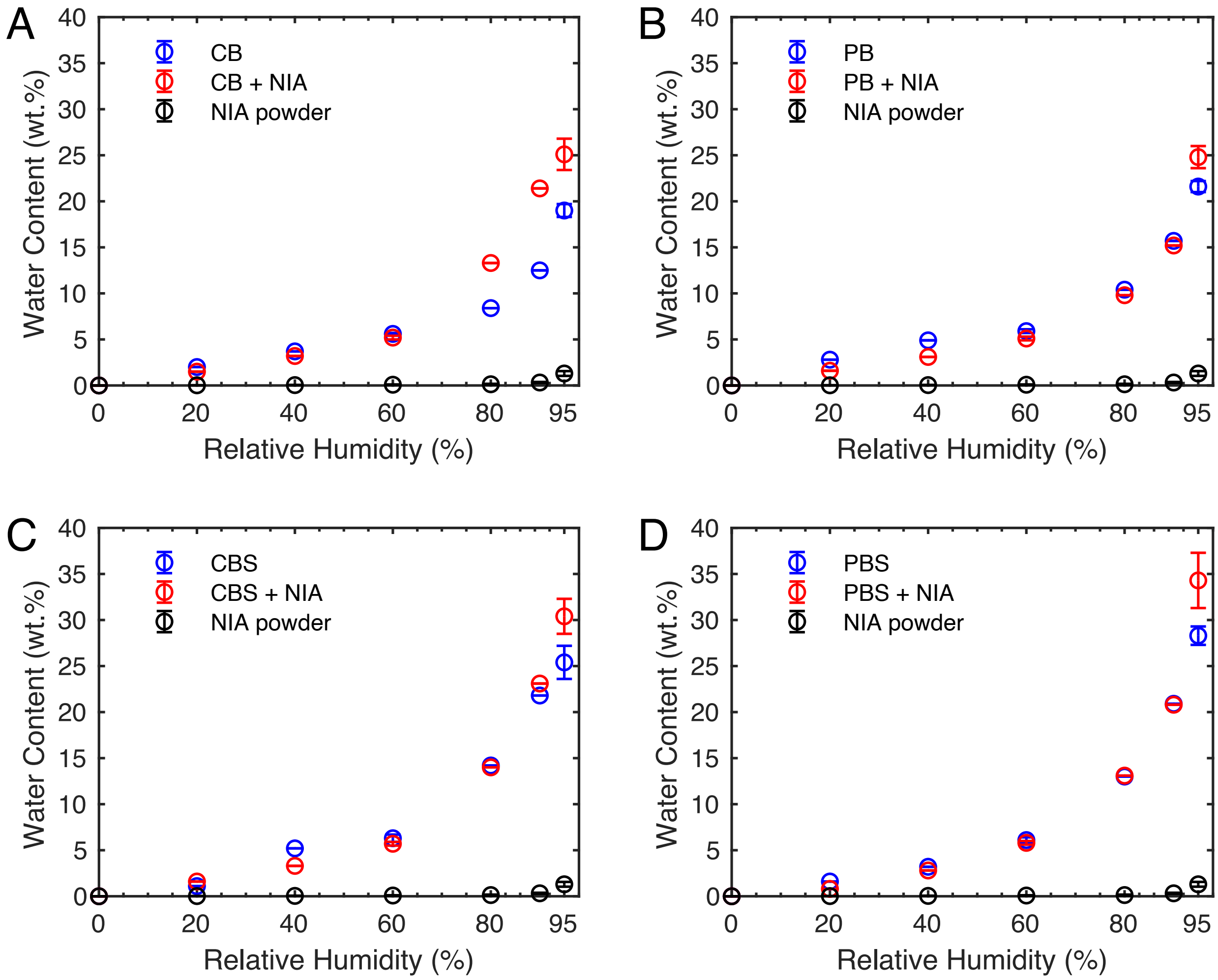

Figure S1. Water sorption isotherms at 25 °C of SC samples pretreated in different buffers. Measurements with SC samples were performed one time for each case and complemented with 5 individual replicates in total for each case at 60% and 95% RH. Niacinamide in powder form (NIA powder) was measured in triplicates. Data with multiple replicates are presented as mean ± SEM (n=5). Abbreviations of pretreatment buffer media: CB – citrate buffer (pH 5.0) (A), PB – phosphate buffer (pH 7.4) (B), CBS – citrate buffer saline (pH 5.0) (C), PBS – phosphate buffer saline (pH 7.4) (D).

Supportive Information
Niacinamide and Its Impact on Stratum Corneum Hydration and Structure

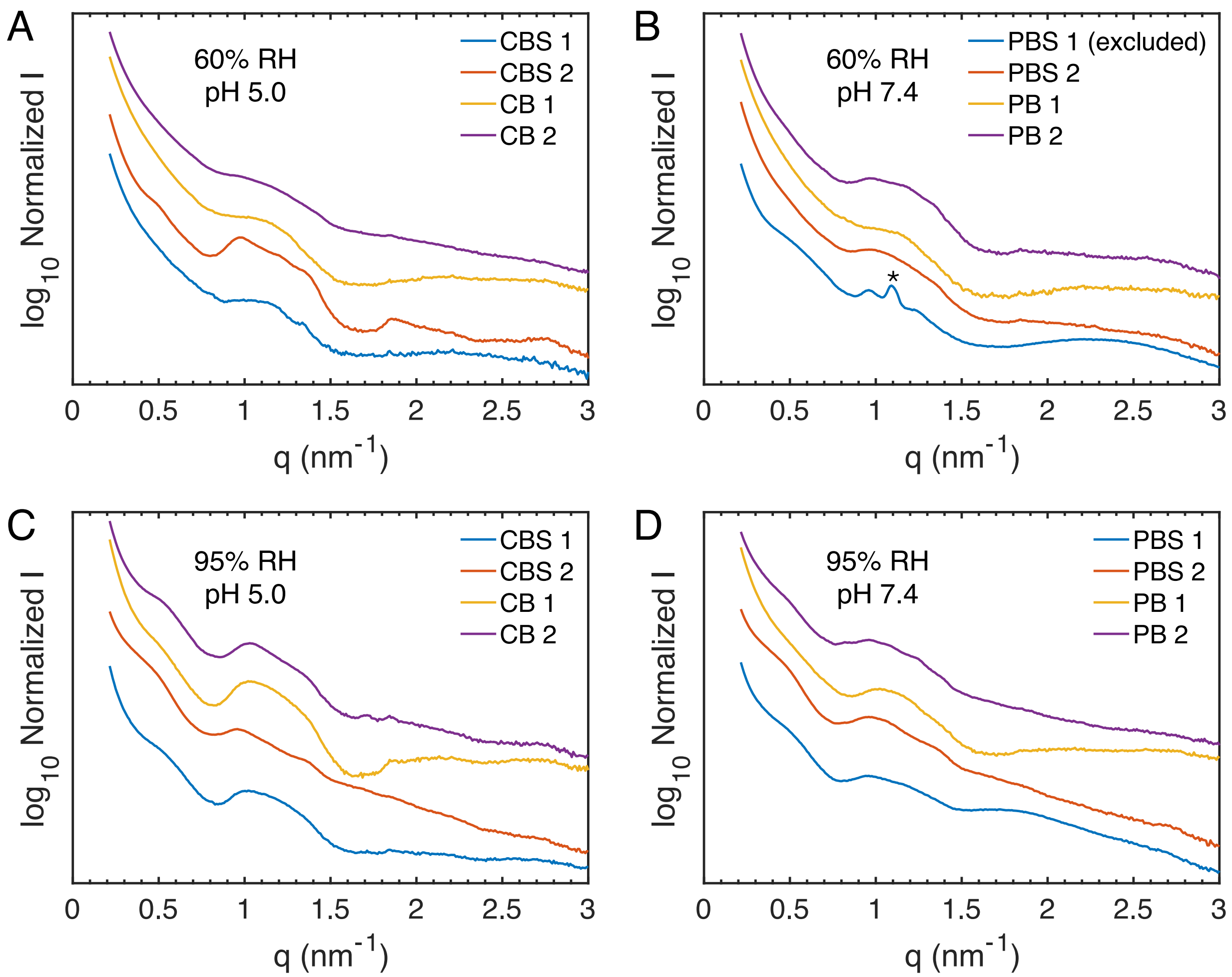

Figure S2. Individual SAXD diffraction curves from SC samples immersed into citrate buffer with or without salt (i.e., CBS or CB, pH 5.0) or phosphate buffer with or without salt (i.e., PBS or PB, pH7.4) for 24 h and then equilibrated at 60% or 95% RH. The normalized diffraction curves are shifted on the y-axis for improved visibility. Note that all diffraction curves correspond to different SC samples (e.g., PBS 1 equilibrated at 60% RH is not the same as the PBS 1 sample equilibrated at 95% RH). The observed biological variation between samples is pronounced, making it difficult identify any effects due to different pH values or presence or absence of salt. On the other hand, the hump occurring around q=0.5 nm^{-1} is more frequently observed at high humidity (i.e., 95% RH) compared to low humidity (i.e., 60% RH). This motivated us to average the intensity values from the diffraction curves in (A) and (B) and compare with the average intensity values from the curves in (C) and (D), as shown in Fig. 3A in the main article. The asterisk in (B) marks a peak with unusually high intensity, motivating the exclusion of this diffraction curve from the average curve presented in Fig. 3A (i.e., n=7 at 60% RH).

Supportive Information
Niacinamide and Its Impact on Stratum Corneum Hydration and Structure

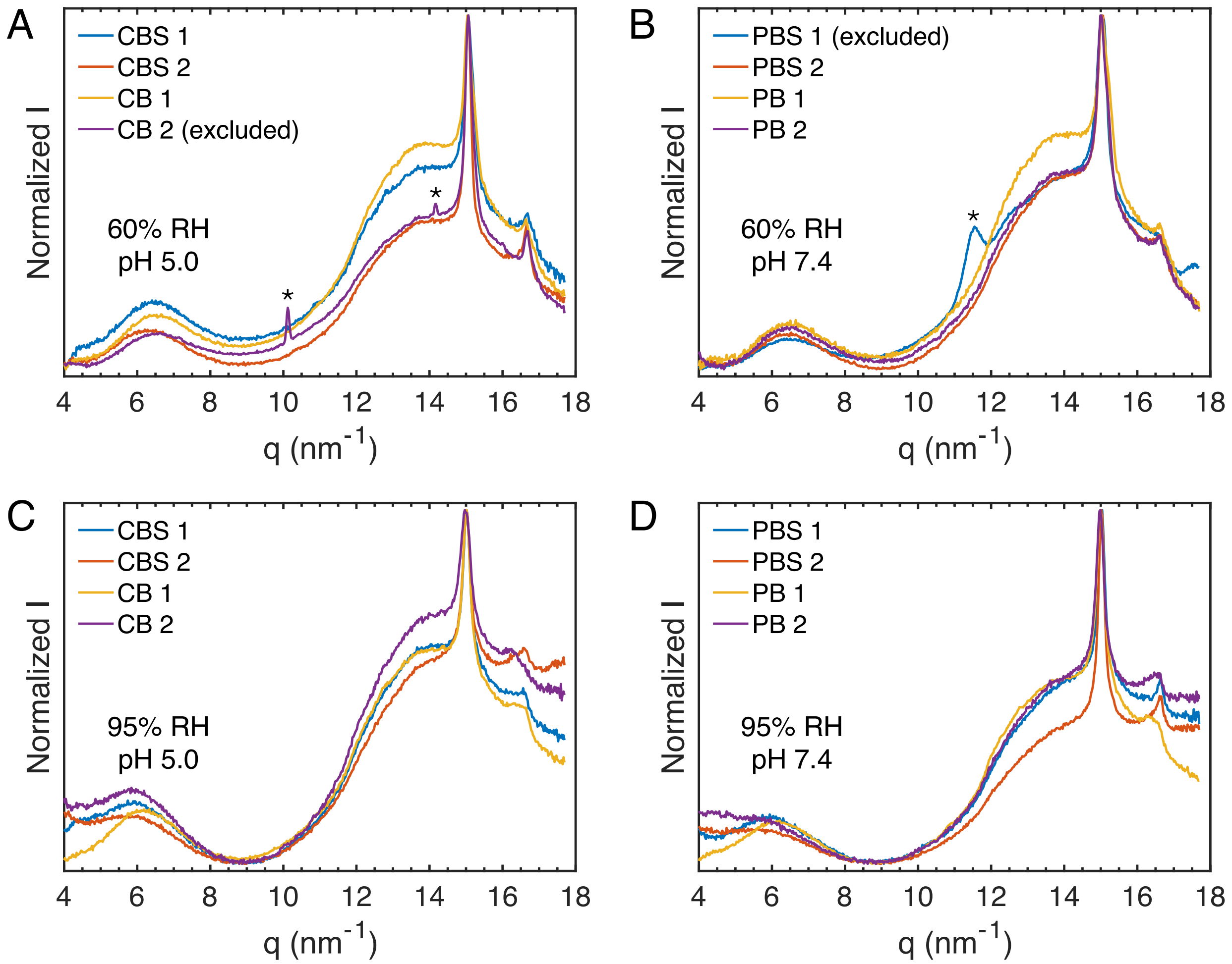

Figure S3. Individual WAXD diffraction curves from SC samples immersed into citrate buffer with or without salt (i.e., CBS or CB, pH 5.0) or phosphate buffer with or without salt (i.e., PBS or PB, pH7.4) for 24 h and then equilibrated at 60% or 95% RH. Note that all diffraction curves correspond to different SC samples (e.g., PBS 1 equilibrated at 60% RH is not the same as the PBS 1 sample equilibrated at 95% RH). The biological variation between samples is less pronounced in the WAXD data as compared to the SAXD data. However, no clear effects are observed due to different pH values or presence or absence of salt. In contrast, the broad hump centered around q=6 nm^{-1} is clearly shifted to lower q-values at high humidity (i.e., 95% RH) compared to low humidity (i.e., 60% RH). This motivated us to average the intensity values from the diffraction curves in (A) and (B) and compare with the average intensity values from the curves in (C) and (D), as shown in Fig. 3B in the main article. The asterisks in (A) and (B) mark peaks with unusually high intensities, motivating the exclusion of these diffraction curves from the average curve presented in Fig. 3B (i.e., n=6 at 60% RH).

Supportive Information
Niacinamide and Its Impact on Stratum Corneum Hydration and Structure

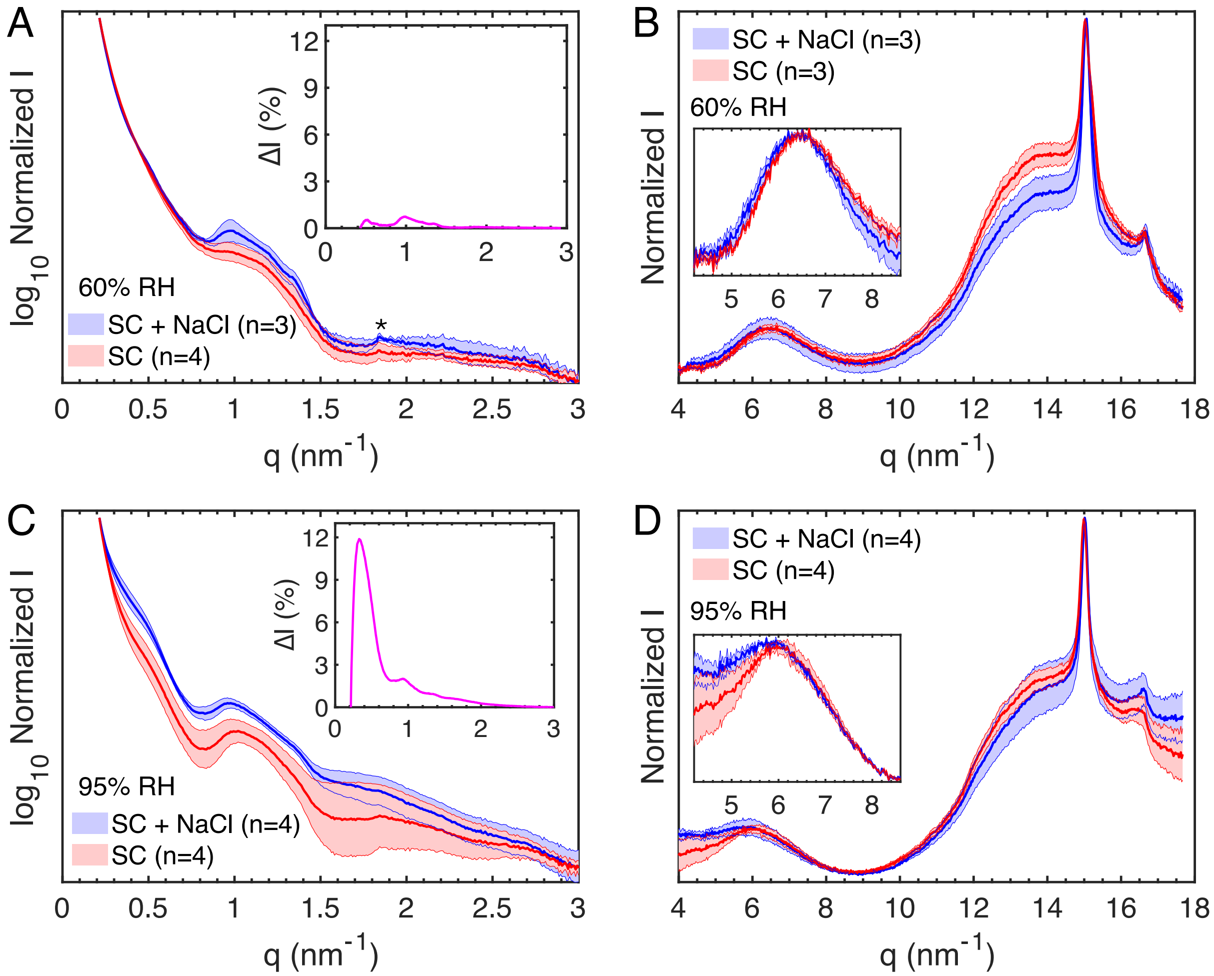

Figure S4. Comparison between SC samples equilibrated at 60% (A and B) and 95% RH (C and D) after pretreatment in buffers without salt and saline buffers (in all cases without NIA). The water contents at 60% RH were 6.2±0.2 and 5.8±0.1 wt.% for SC + NaCl and SC, respectively. The corresponding water contents at 95 % RH were 26.8±1.1 and 20.3±0.5 wt.% for SC + NaCl and SC, respectively. $\Delta I = I_n^{SC+NaCl} - I_n^{SC}$. The asterisks in (A) indicate phase-separated domains of solid cholesterol.

Supportive Information
Niacinamide and Its Impact on Stratum Corneum Hydration and Structure

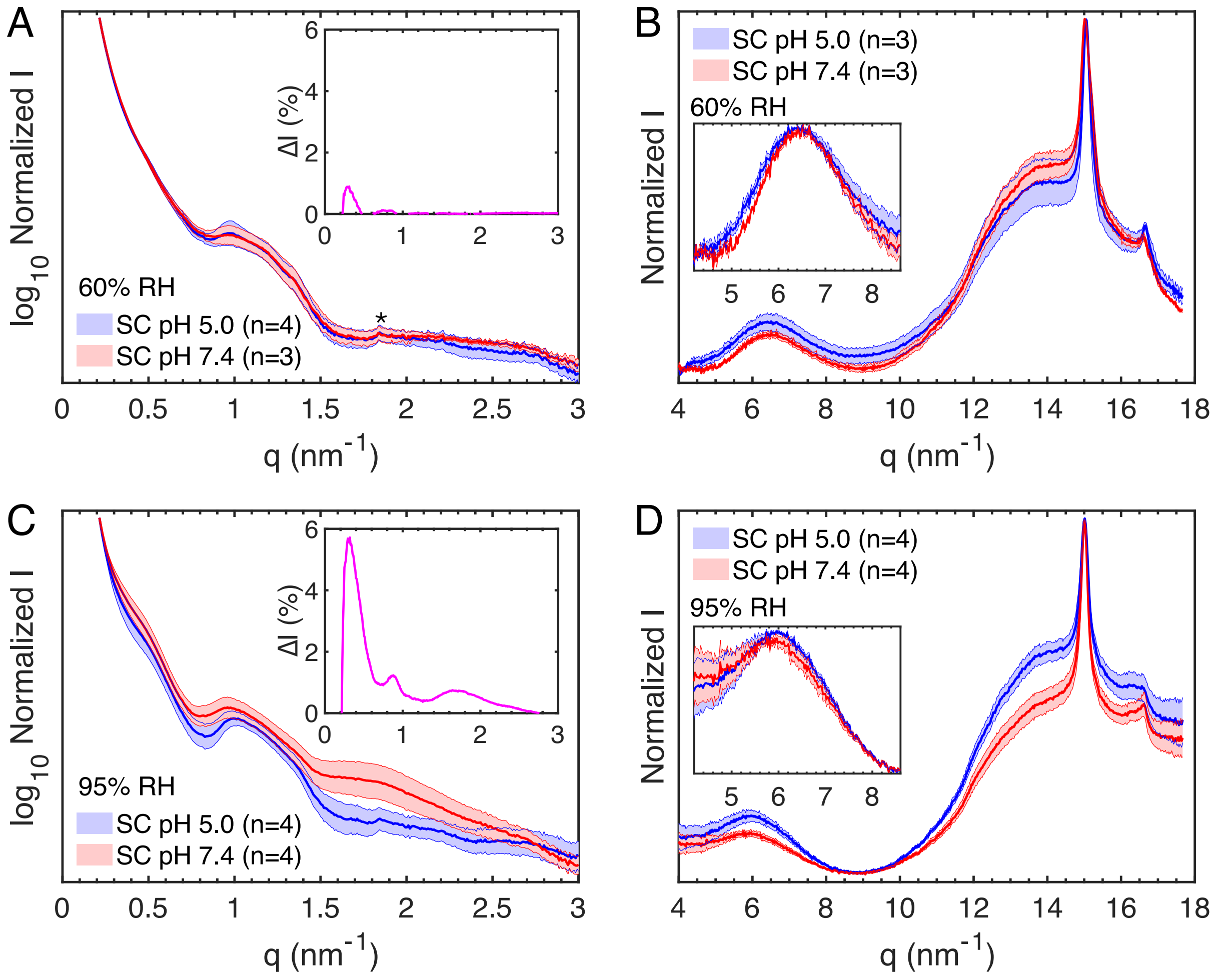

Figure S5. Comparison between SC samples equilibrated at 60% RH (A and B) and 95% RH (C and D) after pretreatment in citrate or phosphate buffers (in all cases without NIA). The water contents at 60 % RH were 5.9±0.2 and 6.0±0.2 wt.% for SC pH 5.0 and SC pH 7.4, respectively. The corresponding water contents at 95 % RH were 22.2±1.4 and 25.0±1.2 wt.% for SC pH 5.0 and SC pH 7.4, respectively. $\Delta I = I_n^{pH\ 7.4} - I_n^{pH\ 5.0}$. The asterisks in (A) indicate phase-separated domains of solid cholesterol.

Supportive Information
Niacinamide and Its Impact on Stratum Corneum Hydration and Structure

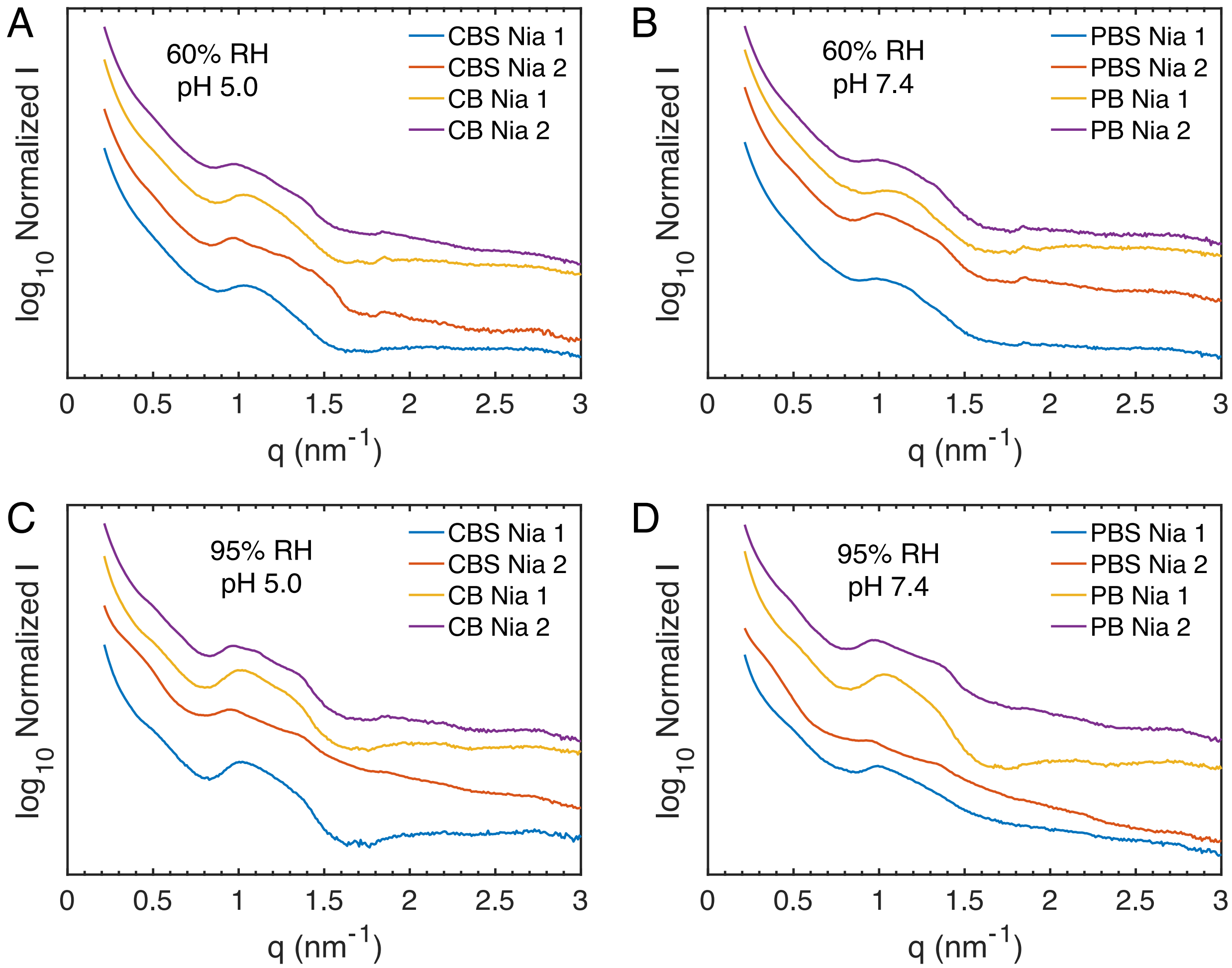

Figure S6. Individual SAXD diffraction curves from SC samples immersed in 5wt% NIA solutions of citrate buffer with or without salt (i.e., CBS or CB, pH 5.0) or phosphate buffer with or without salt (i.e., PBS or PB, pH7.4) for 24 h and then equilibrated at 60% RH (A and B) or 95% RH (C and D). The normalized diffraction curves are shifted on the y-axis for improved visibility. Note that all diffraction curves correspond to different SC samples (e.g., PBS NIA 1 equilibrated at 60% RH is not the same as the PBS NIA 1 sample equilibrated at 95% RH). These data sets represent the data corresponding to NIA treated samples with averaged intensity presented in Fig. 4 in the main article.

Supportive Information
Niacinamide and Its Impact on Stratum Corneum Hydration and Structure

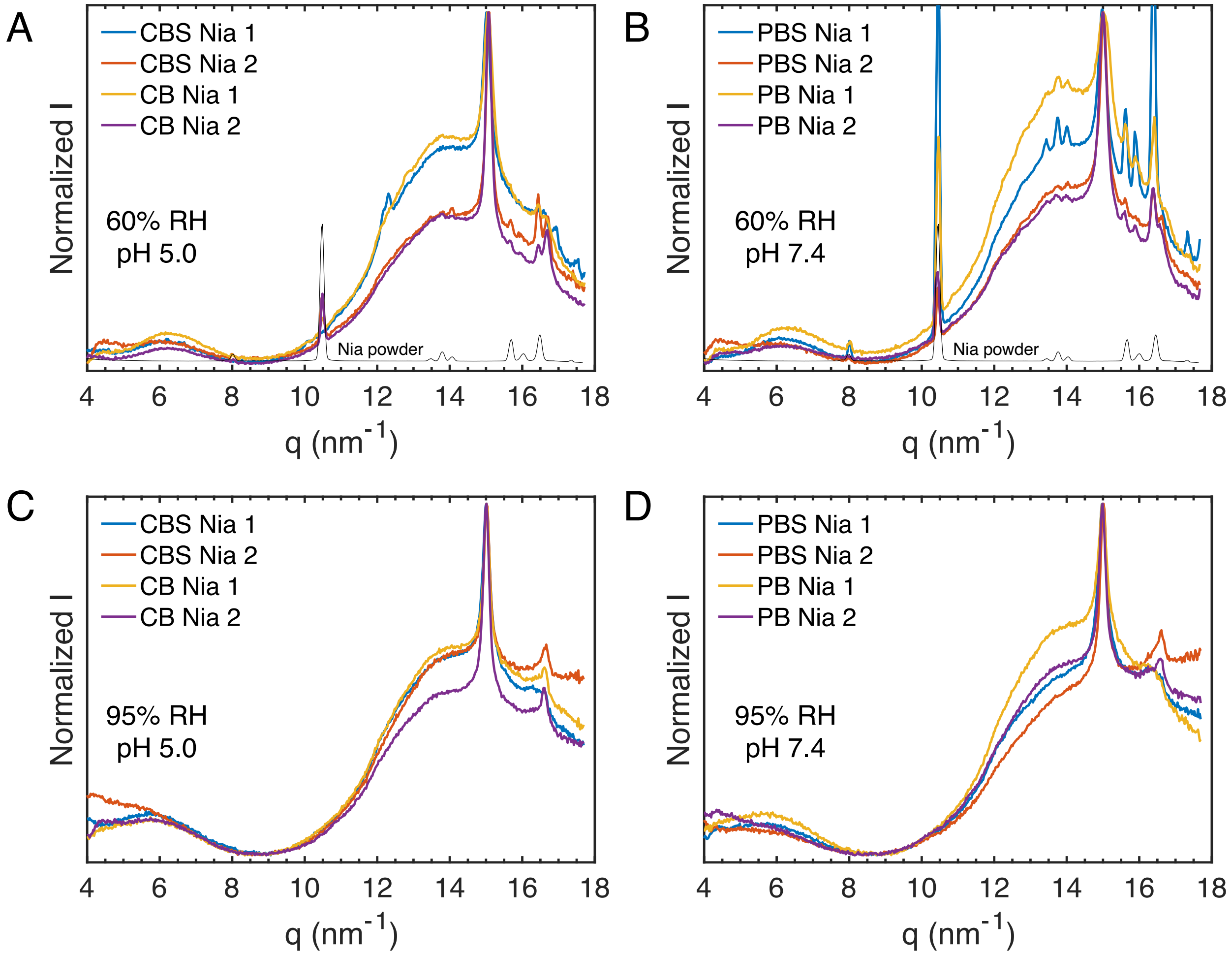

Figure S7. Individual WAXD diffraction curves from SC samples immersed in 5wt% NIA solutions of citrate buffer with or without salt (i.e., CBS or CB, pH 5.0) or phosphate buffer with or without salt (i.e., PBS or PB, pH 7.4) for 24 h and then equilibrated at 60% RH (A and B) or 95% RH (C and D). Note that all diffraction curves correspond to different SC samples (e.g., PBS NIA 1 equilibrated at 60% RH is not the same as the PBS NIA 1 sample equilibrated at 95% RH). In (B), the normalized intensity from PBS NIA 1 was adjusted so that the peak corresponding to hexagonally packed acyl chains was equal to unity to avoid erroneous normalization due to the strong diffraction peaks from phase separated crystalline NIA. These data sets represent the data corresponding to NIA treated samples with averaged intensity presented in Fig. 5 in the main article.

Supportive Information
Niacinamide and Its Impact on Stratum Corneum Hydration and Structure

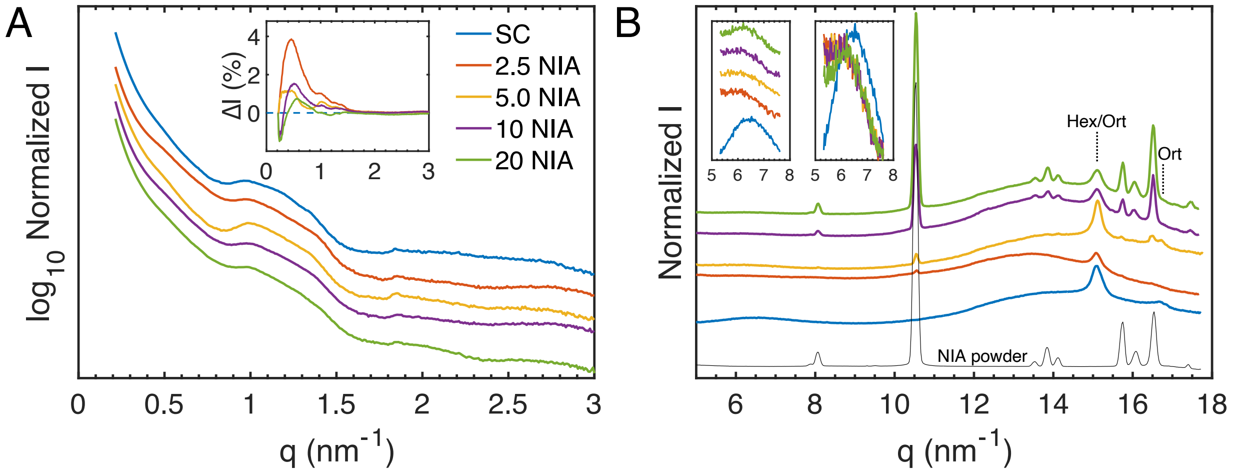

Figure S8. SAXD (A) and WAXD (B) data from untreated SC and SC sheets soaked in PBS (pH 7.4) containing increasing concentrations of NIA (numbers give concentrations in wt.%) for 24h and subsequently equilibrated in 60% RH. Inserts in (A) show the difference in normalized intensity between the diffraction curves obtained from NIA treated samples and control (i.e., $\Delta I = I_n^{SC+NIA} - I_n^{SC}$). The intensity of the WAXD data in (B) was normalized between q = 0.48-17.67 nm^{-1} to dampen the strong intensity from phase separated crystalline NIA. Inserts in (B) show close-ups of the broad diffraction peak from soft keratin filaments (left show raw data of intensity and right show normalized intensity).

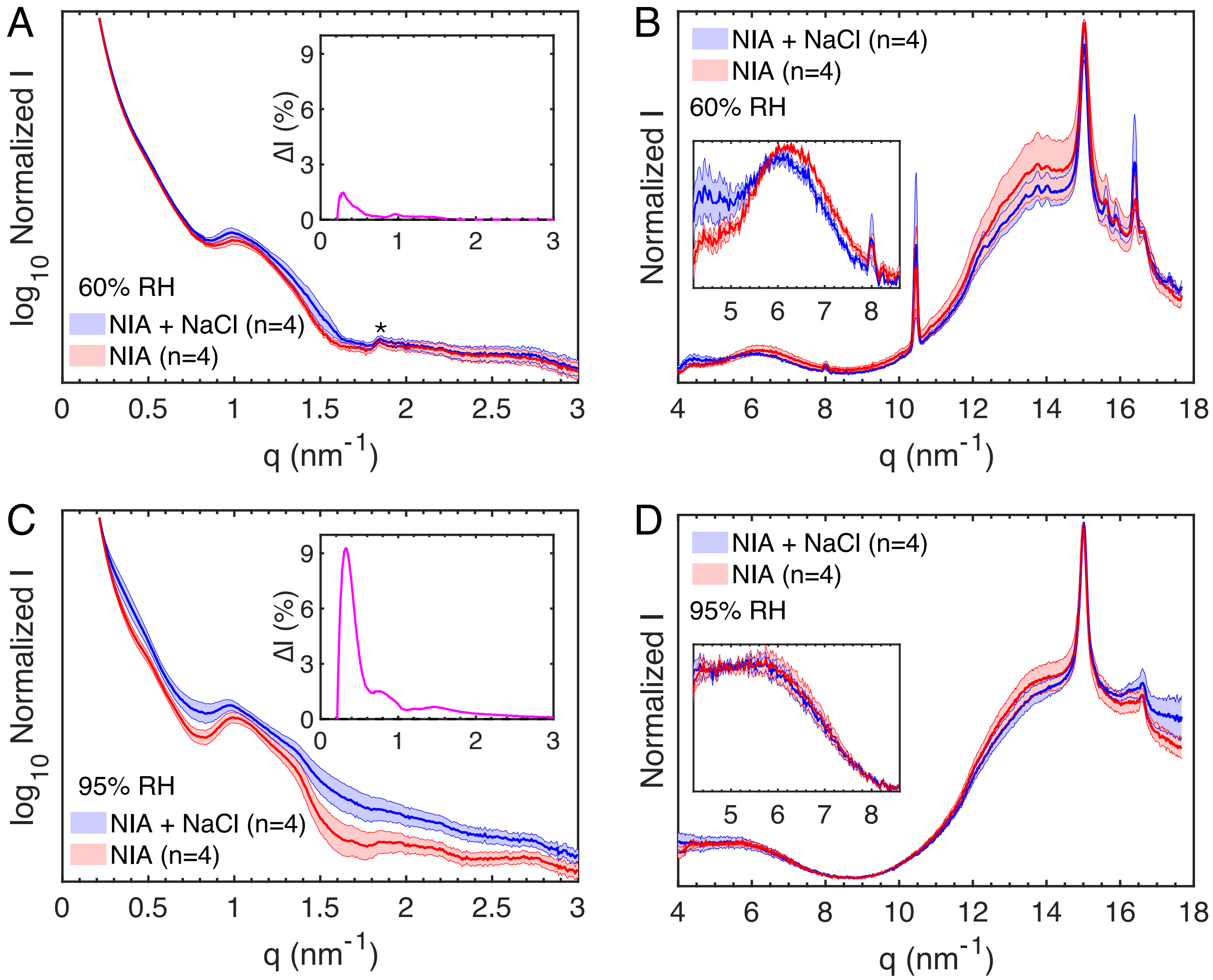

Figure S9. Comparison between SC samples equilibrated at 60% RH (A and B) and 95% RH (C and D) after pretreatment in buffers without salt and saline buffers (in all cases with 5 wt.% NIA). The water contents at 60 % RH were 5.7±0.1 and 5.2±0.1 wt.% for NIA + salt and NIA, respectively. The corresponding water contents at 95 % RH were 32.3±1.8 and 24.9±0.6 wt.% for NIA + salt and NIA, respectively. $\Delta I = I_n^{NIA+NaCl} - I_n^{NIA}$. The asterisks in (A) indicate phase-separated domains of solid cholesterol.

Supportive Information
Niacinamide and Its Impact on Stratum Corneum Hydration and Structure

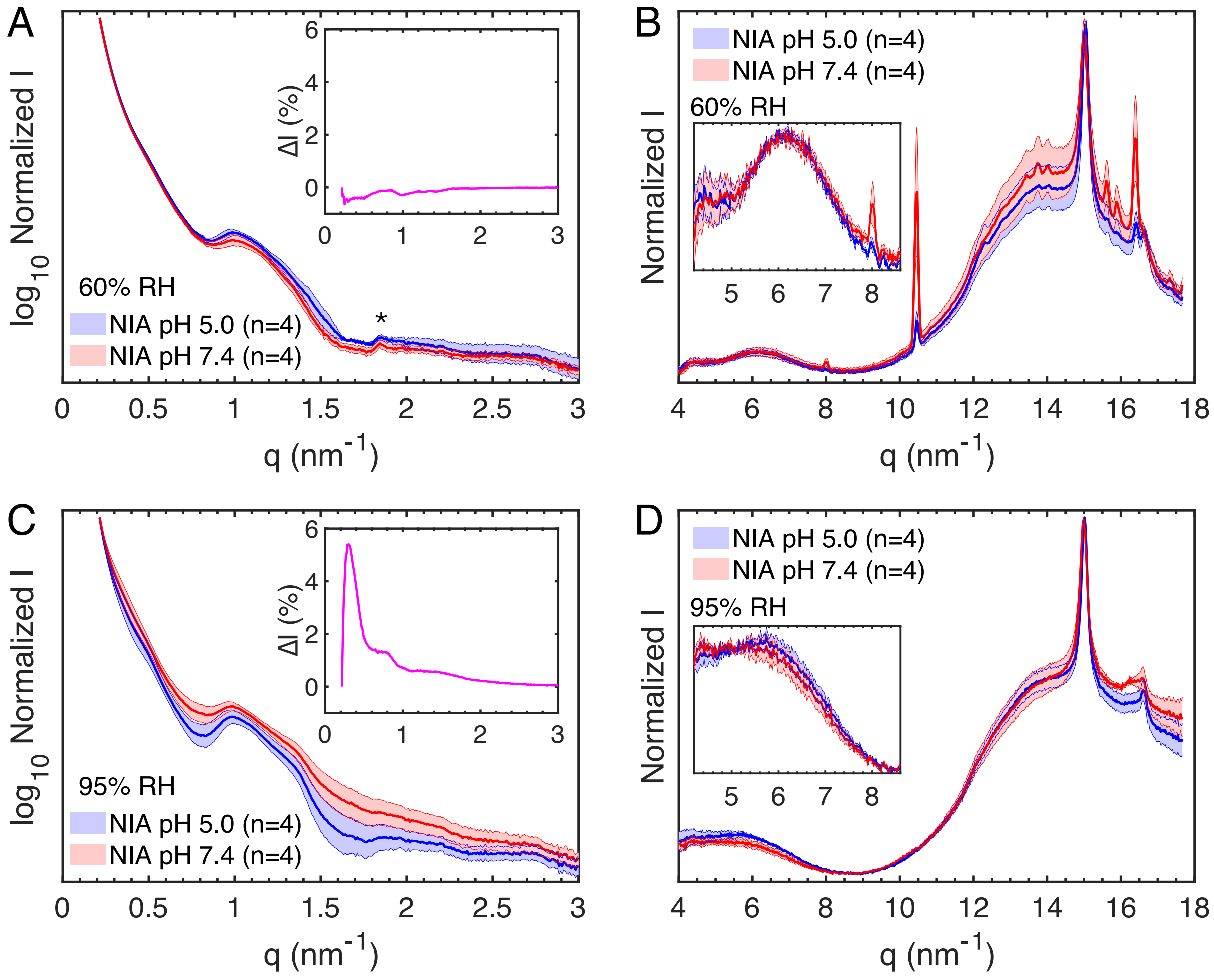

Figure S10. Comparison between SC samples equilibrated at 60% RH (A and B) and 95% RH (C and D) after pretreatment in citrate or phosphate buffers (in all cases with 5 wt.% NIA). The water contents, based on DVS measurements, were 5.4±0.2 and 5.5±0.2 wt.% for NIA pH 5.0 and NIA pH 7.4, respectively, at 60% RH. The corresponding water contents at 95 % RH were 27.7±1.5 and 29.5±2.2 wt.% for NIA pH 5.0 and NIA pH 7.4, respectively. $\Delta I = I_n^{NIA\ pH\ 7.4} - I_n^{NIA\ pH\ 5.0}$. The asterisks in (A) indicate phase-separated domains of solid cholesterol.

Supportive Information
Niacinamide and Its Impact on Stratum Corneum Hydration and Structure

Supportive Tables

Table S1. Statistical analysis of water sorption data comparing the effects of buffers without NIA and buffers containing 5 wt.% NIA. Compilation of *p*-values for one-tailed independent t-tests assuming equal variances.

Condition	CB (pH 5, no salt)	CBS (pH 5, extra salt)	PB (pH 7.4, no salt)	PBS (pH 7.4, extra salt)
Comparison	No NIA VS NIA	No NIA VS NIA	No NIA VS NIA	No NIA VS NIA
60% RH	0.0790	0.1157	0.0183 (*)	0.1504
95% RH	0.0053 (**)	0.0448 (*)	0.0231 (*)	0.0620

Table S2. Statistical analysis of water sorption data comparing the effects of buffers without extra salt and saline buffers. Compilation of *p*-values for one-tailed independent t-tests assuming equal variances.

Condition	CB (pH 5)	CB (pH 5 + NIA)	PB (pH 7.4)	PB (pH 7.4 + NIA)
Comparison	No salt VS Extra salt	No salt VS Extra salt	No salt VS Extra salt	No salt VS Extra salt
60% RH	0.0850	0.0575	0.2579	0.0172 (*)
95% RH	0.0053 (**)	0.0357 (*)	0.0002 (***)	0.0098 (**)

Table S3. Statistical analysis of water sorption data comparing the effects of acidic and neutral buffers. Compilation of *p*-values for one-tailed independent t-tests assuming equal variances.

Condition	No salt (CB or PB)	Extra salt (PBS or CBS)	No salt + NIA (CB or PB)	Salt + NIA (PBS or CBS)
Comparison	pH 5.0 VS pH 7.4	pH 5.0 VS pH 7.4	pH 5.0 VS pH 7.4	pH 5.0 VS pH 7.4
60% RH	0.1939	0.3927	0.4273	0.3894
95% RH	0.0117 (*)	0.0933	0.4377	0.1536
